# Nicotine attenuates D-galactose- induced hippocampal aging-like alterations with α7-nAChR- associated mechanisms

**DOI:** 10.3389/fnagi.2026.1947643

**Published:** 2026-10-19

**Authors:** Xin Li, Yue Li, Yingyan Li, Wenming Wang, Shuhao Ma, Jun Li, Xuan Cheng, Chunmei Guang, Enliang Hong, Huan Chen, Hongwei Hou, Qingyuan Hu

**Affiliations:** 1 Beijing Life Science Academy, Beijing, China; 2 Key Laboratory of Tobacco Biological Effects, Zhengzhou, China; 3 China National Tobacco Quality Supervision and Test Center, Zhengzhou, China

**Keywords:** cognitive impairment, D-galactose, hippocampus, neuroinflammation, nicotine, oxidative stress, senescence, α7 nicotinic acetylcholine receptor

## Abstract

**Background:**

Chronic D-galactose exposure produces selected behavioral and cellular changes associated with aging. We examined whether nicotine attenuates these changes and whether its effects differ between wild-type rats and α7 nicotinic acetylcholine receptor (α7-nAChR) knockout rats.

**Methods:**

Female and male rats received saline, D-galactose (180 mg/kg/day), or D-galactose plus nicotine (0.5 mg/kg/day) for 49 days. We assessed spatial behavior, hippocampal staining and gene expression, and peripheral biochemical measures. Nineteen animal endpoints were analyzed using sex × genotype × treatment models, with multiplicity-adjusted contrasts and unequal-variance sensitivity analyses. Exploratory hippocampal RNA sequencing included six wild-type rats per sex and treatment.

**Results:**

Compared with wild-type rats receiving D-galactose alone, nicotine-co-treated wild-type rats had shorter latency to first target entry, lower mean distance to the target, and more platform-site crossings in the water maze. They also had higher serum reduced glutathione (GSH) and lower hippocampal astrocytic and microglial immunoreactivity. The nicotine-associated increases in platform-site crossings and serum GSH, and the reduction in hippocampal ionized calcium-binding adapter molecule 1 (IBA1) immunoreactivity, were greater in wild-type than knockout rats. These differences were supported by both variance analyses. Nicotine co-treatment was also associated with reduced senescence-associated β-galactosidase staining and lower hippocampal *App*, *Bace1*, and *Psen1* mRNA levels in wild-type rats. Serum amyloid-β1–42 did not change significantly. RNA sequencing identified treatment-associated expression patterns in wild-type hippocampi.

**Conclusion:**

Nicotine attenuated selected D-galactose-associated behavioral and hippocampal alterations. The genotype comparisons support α7-nAChR-associated modulation of platform-site crossings, serum GSH, and hippocampal IBA1. They do not establish that changes in brain GSH or amyloid production mediate these effects, or that all nicotine responses share one receptor-dependent mechanism.

## 1 Introduction

Brain aging is accompanied by changes in redox balance, mitochondrial function, immune regulation, synaptic plasticity, and cellular stress responses. Chronic D-galactose exposure is used to investigate selected aspects of this biology over a defined experimental period. Rodent studies have reported learning deficits, oxidative stress, and inflammatory or senescence-associated changes after D-galactose treatment (Wei et al., 2005; Wang et al., 2009; Zhang et al., 2021; Jeong et al., 2021). These processes are relevant to aging, but the exposure model does not reproduce the time course or full pathology of natural aging or Alzheimer’s disease. Mitochondrial oxidative stress and senescence-associated inflammation provide complementary biological contexts for interpreting its effects (Paradies et al., 2011; Cai et al., 2020).

A practical advantage of the D-galactose model is that it allows selected stress responses to be examined within a shorter period than natural-aging studies. This advantage must be balanced against dose-, route-, duration-, and sex-dependent effects and possible systemic toxicity. Recent rat studies illustrate this variability, reporting cognitive, mitochondrial, inflammatory, and other aging-associated changes under different exposure conditions (Martinovic et al., 2023; Pantiya et al., 2023; Younis et al., 2024; Medeiros et al., 2025). We therefore use the model as a controlled aging-like challenge, not as evidence of established Alzheimer-type pathology.

Nicotinic acetylcholine receptors (nAChRs) influence neuronal excitability, intracellular calcium signaling, and neuroimmune communication. The α7 subtype is of interest because it is expressed in neuronal and glial systems and can modulate oxidative and inflammatory responses (Fontana et al., 2023; Liu and Zhao, 2004; Liu et al., 2015; Parada et al., 2010; Piovesana et al., 2021; Hurst et al., 2013). Nicotine activates several nAChR subtypes, so its effects cannot automatically be assigned to α7-nAChR (Hurst et al., 2013; Terry and Callahan, 2019). Experimental studies have linked α7-selective stimulation to reduced inflammatory signaling and have shown that nicotine can suppress stimulus-induced astrocyte activation (Park et al., 2022; Cao et al., 2024). Functional α7-mediated calcium responses have also been reported in human induced pluripotent stem cell-derived astrocytes, without establishing neuroprotection or long-term safety (Cöllen et al., 2026).

Here, nicotine serves as an experimental pharmacological probe. Its dependence liability and other systemic risks preclude interpreting an experimental benefit as support for nicotine or tobacco consumption. Receptor-selective ligands and endogenous cholinergic agonists provide complementary approaches for investigating receptor biology without making that inference.

Majdi et al. (2018) reported improved memory and reduced mitochondrial oxidative damage and apoptosis in D-galactose-treated mice given nicotine. Zhang et al. (2023) subsequently showed that the α7-nAChR agonist PNU282987 attenuated cognitive, oxidative, inflammatory, and senescence-associated abnormalities in D-galactose-treated rats and BV2 cells, with evidence implicating Nrf2/HO-1 signaling. These studies provide a rationale for testing whether nicotine responses differ between wild-type and α7-nAChR knockout animals.

Nicotine may also act through mechanisms that are not restricted to classical nAChR signaling. Yang et al. (2023) reported enhanced NAMPT activity and improved NAD+ homeostasis in aging male mice, whereas Jia et al. (2025) reported altered glycolipid and sphingolipid metabolism during prolonged oral nicotine exposure. These findings motivate consideration of both receptor-associated and broader metabolic responses; they do not establish that the same mechanisms operate in the present model.

We hypothesized that nicotine co-treatment would attenuate selected D-galactose-associated behavioral, inflammatory, and glial alterations, and that some treatment responses would differ according to α7-nAChR genotype. The key mechanistic question was whether the change produced by nicotine differed directly between genotypes, rather than whether a comparison was significant in wild-type rats but not in knockout rats. Developmental compensation and other cohort differences remain relevant to this interpretation.

We also examined hippocampal App, Bace1, and Psen1 transcripts as exploratory markers of amyloid precursor protein (APP) processing. APP supplies the precursor substrate, BACE1 initiates its amyloidogenic cleavage, and presenilin-1 is a component of the γ-secretase machinery (Vassar et al., 1999; De Strooper et al., 1998). These measurements asked whether nicotine-associated changes in the aging-like phenotype were accompanied by changes in this transcriptional panel. They were not designed to substitute for measurements of the corresponding proteins, secretase activity, or brain amyloid burden. Serum GSH and other peripheral measures provided a separate assessment of the systemic response.

We tested this hypothesis in female and male wild-type and α7-nAChR knockout rats on a Sprague–Dawley background. Behavioral and hippocampal outcomes were interpreted alongside, but not equated with, peripheral biochemical measures. An exploratory transcriptomic analysis in wild-type hippocampi provided additional pathway context without testing receptor dependence.

## 2 Materials and methods

### 2.1 Animals, housing, and experimental design

Twelve-week-old wild-type (WT) rats and α7-nAChR knockout (α7-KO) rats were used. WT rats were obtained from Zhejiang Vital River Laboratory Animal Technology Co., Ltd. (license SCXK [Zhe] 2019-0001), whereas the *Chrna7* knockout rat model was generated by Shanghai Model Organisms Center, Inc. (Shanghai, China; laboratory animal production license SCXK [Hu] 2024-0007). Both cohorts were on a Sprague–Dawley genetic background. Male and female animals were included in equal proportions, and genotypes were confirmed before group allocation. Animals were maintained in a barrier facility at 20–26 °C, with daily temperature variation ≤4 °C, relative humidity of 40–70%, a 12:12-h light/dark cycle, illuminance of 15–20 lux, and ad libitum access to food and water.

After one week of acclimation, animals within each genotype cohort were randomly allocated to the Control, D-galactose Model, or D-galactose plus Nicotine group. D-galactose was administered at 180 mg/kg/day and nicotine at 0.5 mg/kg/day for 49 consecutive days, with daily dosing scheduled at 10:30. Nicotine was used in its free-base form, and the nicotine dose is expressed as free base. Both compounds were dissolved in normal saline. In the combined-treatment group, D-galactose and nicotine were mixed and administered together as a single daily intraperitoneal injection. Model animals received D-galactose in normal saline, and Control animals received an equivalent volume of normal saline.

The D-galactose stock solution contained 5 g in 30 mL normal saline. For each rat, the required amounts of D-galactose and, where applicable, nicotine were calculated from body weight, and normal saline was added to a final injection volume of 1 mL per rat per administration. The nicotine stock solution was prepared at 1 mg/mL, aliquoted, stored at −80 °C, and passed through a 0.22-μm sterile filter before administration. The same nicotine reagent was used in the animal and cell-culture experiments.

The final administration was performed in the morning, and water-maze training began the following morning, approximately 24 h later. Treatment was not continued during behavioral testing. After testing, animals were anesthetized with sodium pentobarbital for terminal sample collection; euthanasia was completed by cervical dislocation under anesthesia. Serum, urine, brain tissue, and hippocampal samples were collected for subsequent analyses.

The number of animals analyzed for each endpoint is indicated in the corresponding figure legends and is represented by the individual data points shown in each figure.

### 2.2 PCR genotyping of α7-nAChR knockout rats

The *Chrna7* knockout model was generated using CRISPR/Cas9 targeting exons 4 and 5 of transcript *Chrna7*-203 (ENSRNOT00000051883.5), as depicted in Supplementary Figure 1A. Genotyping primer sequences are provided in the Supplementary Figure 1 legend. Genotypes were assessed before group allocation by allele-specific PCR. The expected P1/P2 products are 1756 bp for the wild-type allele and 601 bp for the knockout allele; P3/P4 is expected to produce a 284-bp wild-type product and no corresponding knockout product. Supplementary Figure 1C,D show separate sets of animals, with P1/P2 reactions above and P3/P4 reactions below. These assays identify the targeted genomic allele; they do not measure hippocampal receptor protein abundance or function. A transcript assay outside the deleted region could detect retained RNA without demonstrating an intact functional receptor; residual *Chrna7* qPCR signal is therefore not interpreted as evidence of an intact receptor. The receptor transcript is designated *Chrna7* (α7-nAChR).

### 2.3 Morris water maze

Spatial learning and memory were evaluated using a circular water tank with an 80-cm radius and 45-cm wall height. Water temperature was maintained at approximately 25 °C. The tank was divided into four quadrants, and a circular platform 12 cm in diameter was positioned at a fixed location 2.5 cm below the water surface. Stable distal visual cues were placed around the apparatus (Dyomina et al., 2023).

Water-maze training began approximately 24 h after the final drug administration. The place-navigation phase comprised four trials per day for five consecutive days, with an interval of approximately 1 h between trials. Starting quadrants were varied across trials. Rats were allowed up to 90 s to locate the hidden platform and remained on the platform for 30 s after successful escape. Behavior was recorded using Smart 3 tracking software. On day 6, the platform was removed for a 120-s probe trial, during which platform-site crossings were recorded. The animal-level outcomes reported in Figure 2 comprise latency to first target entry, mean distance to target, and platform-site crossings. Mean distance to target was treated as a distinct endpoint rather than as total swim-path length. The treatment and testing schedule is shown in Figure 2A. The acquisition phase and the platform-free probe trial assess related but distinct aspects of spatial performance (Vorhees and Williams, 2006).

### 2.4 Oxidative stress and peripheral biomarker measurements

Superoxide dismutase (SOD) and malondialdehyde (MDA) were measured in rat serum using assay kits Ml059387 and ml640319, respectively, from Shanghai Enzyme-linked Biotechnology Co., Ltd. (Shanghai, China). SOD was reported as concentration in ng/mL, not enzymatic activity; MDA was reported in nmol/mL. Reduced glutathione (GSH) was measured in rat serum using kit ML531010 from the same supplier and was reported in mmol/L. Serum IL-1β, IL-6 and TNF-α were measured by enzyme-linked immunosorbent assay (ELISA); the respective kit identifiers were Ml003057, Ml064292 and Ml002859 from the same supplier. Serum ApoE, total Tau and Aβ1–42 were measured using commercial ELISA kits from the same supplier; the ApoE, Tau and Aβ1–42 kit identifiers were ml003325, ml038138 and ml003356, respectively. Samples were incubated in antibody-coated microplates for 1 h at 37 °C, washed five times, and read at 450 nm using a TECAN microplate reader. Supplementary Figures 2–4 display fold values relative to the same-sex WT Control mean, as defined in Methods 2.10; the underlying measurements and original units are retained in the source-data file and Supplementary Table P7. GSH was not measured in brain tissue; the serum assay is interpreted as a peripheral biochemical measure rather than a surrogate for hippocampal GSH.

Urinary formate was measured using a formic acid assay kit (ab111748; Abcam, Cambridge, UK). Ten microliters of urine were loaded per well, absorbance was measured at 450 nm, and values were calculated from the standard curve using a SpectraMax Paradigm Multi-Mode reader (Molecular Devices, San Jose, CA, USA). Unscaled assay values are provided in Supplementary Data 2. Supplementary Figure 4D displays dimensionless ratios relative to the same-sex WT Control mean. Urine measurements were not normalized to creatinine or specific gravity. ApoE, total Tau, Aβ1–42, and urinary formate were treated as exploratory peripheral outcomes rather than direct measures of hippocampal pathology.

### 2.5 Histopathology, immunohistochemistry, and TUNEL staining

Brain and other collected tissues were fixed in 10% formalin, embedded in paraffin, sectioned at 5 μm using a Leica rotary microtome, and processed for hematoxylin and eosin (H&E) staining. H&E staining included five biological replicates per group and was examined at 200×. For immunohistochemistry, paraffin-embedded hippocampal sections were incubated with antibodies against glial fibrillary acidic protein (GFAP), ionized calcium-binding adapter molecule 1 (IBA1), IL-1β, IL-6, TNF-α, and Aβ, followed by species-matched secondary antibodies and 3,3′-diaminobenzidine chromogenic detection. Sections were counterstained, mounted with neutral resin, and examined using an Olympus light microscope. Five rats per sex, genotype, and treatment contributed one section per rat, with five fields photographed at 200× per section. Measurements from the five fields were combined into one observation for each rat. Statistical comparisons used these animal-level observations, rather than treating fields from the same section as independent samples. GFAP, IBA1, IL-1β, IL-6, and TNF-α immunoreactivity was quantified as integrated optical density (IOD). The antibodies were GFAP 16825-1-AP and IBA1 10904-1-AP from Proteintech, and IL-1β ab283818, IL-6 ab9324, TNF-α ab307164, and Aβ ab201060 from Abcam. All six primary antibodies were used at 1:100. Resource identifiers and study applications are listed in Supplementary Table 1. Representative photomicrographs were resized proportionally for presentation, with image scale bars retained. Supplementary Figures 10–16 provide hippocampal anatomical overviews, separately from the high-magnification quantitative fields.

Apoptotic DNA fragmentation was assessed using an *in situ* terminal deoxynucleotidyl transferase-mediated dUTP nick-end labeling (TUNEL) detection kit (Sangon Biotech). Sections were deparaffinized, rehydrated, and incubated with proteinase K for 25 min at 37 °C. TUNEL signal was visualized with DAB, followed by hematoxylin counterstaining. Positive nuclei were quantified in hippocampal sections.

### 2.6 Hippocampal SA-β-gal staining

For senescence-associated β-galactosidase (SA-β-gal) staining, hippocampal frozen sections were dried at 37 °C for 20–30 min, fixed in β-galactosidase staining fixative for 15 min at room temperature, washed three times with phosphate-buffered saline, and incubated overnight at 37 °C with SA-β-gal staining solution (C0602; Beyotime Biotechnology). SA-β-gal-positive area was quantified from digital images and analyzed at the animal level. Descriptive counts of discernible blue or blue-green cell bodies were also obtained using software-assisted visual image annotation of representative images, excluding label and scale-bar regions. Weak staining, unresolved overlaps, and ambiguous deposits were classified separately as uncertain objects. Six tissue fields and eleven unique culture fields were assessed; the Model field shared between the two culture series was counted once. These field-level counts are presented descriptively in Supplementary Table 10, separately from the animal-level positive-area analysis.

### 2.7 Hippocampal quantitative reverse-transcription PCR (qRT-PCR)

Total RNA was extracted from approximately 100 mg of hippocampal tissue using the TaKaRa MiniBEST Universal RNA Extraction Kit (9767; Takara Bio, Japan). RNA quality and concentration were evaluated by agarose gel electrophoresis and NanoDrop 2000 spectrophotometry. The PrimeScript RT reagent Kit with gDNA Eraser (RR047A; Takara Bio) was used with 1.2 μg total RNA. Genomic DNA removal at 42 °C for 2 min was followed by cDNA synthesis in a final 20-μL reaction at 37 °C for 15 min and inactivation at 85 °C for 5 s. Quantitative PCR was performed on a LightCycler 480 system (Roche, Germany) using TB Green Premix Ex Taq II (Tli RNaseH Plus; RR820A; Takara Bio). Cycling conditions were 95 °C for 5 min, followed by 40 cycles of 95 °C for 10 s, 56 °C for 10 s, and 72 °C for 10 s. *Gapdh* was used as the reference gene, and relative expression was calculated by the comparative Ct method. The target panel comprised *Ache*, *App*, *Bace1*, *Psen1*, and *Chrna7*, which encodes α7-nAChR. *App*, *Bace1*, and *Psen1* measurements represent mRNA abundance; the corresponding protein levels and secretase activities were not assessed. Primer sequences and primer melting temperatures are provided in Supplementary Table 2. Amplification and melting curves are shown in Supplementary Figure 17. Relative-expression analyses assess within-genotype treatment responses; *Chrna7* transcript measurements are distinguished from receptor protein abundance or function.

### 2.8 Primary glial cultures and D-galactose-associated SA-β-gal assay

Mouse brains were collected after decapitation and transferred to pre-chilled dishes containing 2 mL Dulbecco’s modified Eagle medium (DMEM). After removal of the olfactory bulbs, vascular membranes, cerebellum, and interhemispheric connective tissue, cerebral cortices were isolated and digested with 2 mL trypsin at 37 °C for 10 min. Digestion was terminated and the suspension was passed sequentially through 70-μm and 40-μm filters. Cells were seeded in T25 flasks and maintained at 37 °C with 5% CO_2_. After 24 h, non-adherent debris was removed and cultures were washed with phosphate-buffered saline and maintained in 5 mL DMEM containing 10% fetal bovine serum for a further 3 days. The upper layer of round or oval cells was removed, and adherent cells were detached with trypsin, centrifuged at 300×g, resuspended, counted, and plated at 1 × 10^5^ cells per well in 24-well plates. The resulting adherent cultures are referred to as astrocyte-enriched primary glial cultures.

D-galactose (V900922-100G) at 200 mM was used in the nicotine co-exposure experiment. Cells were seeded 24 h before treatment. Nicotine was applied as a 2-h pretreatment, followed by 7 days of exposure to 200 mM D-galactose and nicotine across the tested concentration range. The nicotine-treated conditions comprised 50–200 μM, with untreated and D-galactose Model conditions included as controls. The same free-base nicotine reagent was used in the animal and culture experiments. Cells were fixed for 15 min, washed three times, incubated overnight without CO_2_ in SA-β-gal staining solution (C0602; Beyotime Biotechnology), and imaged in randomly selected fields for ImageJ analysis. For each condition, cells from each independent donor mouse were plated in one well, and that well contributed one data point. Four donor mice therefore provided four independent wells and four data points per condition. Microscopic fields within a well were subsamples of that donor-derived culture. Published astrocyte experiments provide pharmacological context for micromolar nicotine exposure (Aryal et al., 2021; Lombardi et al., 2025; Revathikumar et al., 2016), while mechanistic work has examined astroglial α7-associated inflammatory signaling (Patel et al., 2017). The culture endpoint was SA-β-gal staining. A separate BV2 D-galactose screening dataset is provided descriptively in Supplementary Table 15.

### 2.9 Exploratory hippocampal transcriptomics

Total RNA was extracted from homogenized hippocampal tissue using TRIzol and quantified using a NanoDrop ND-2000 spectrophotometer (Thermo Fisher Scientific, Waltham, MA, USA). RNA integrity was assessed using an Agilent 2100 Bioanalyzer, and samples with an RNA integrity number ≥6.0 were accepted. BGI prepared poly(A)-enriched libraries using oligo(dT) capture, random-N6 reverse transcription, double-stranded DNA synthesis, PCR amplification, and single-stranded circular library preparation. Sequencing was performed on DNBSEQ-T7 with paired-end 150-bp reads. Six independent WT rats were included per sex and treatment, yielding 36 samples. SOAPnuke removed adapter-containing reads, reads with more than 0.1% unknown bases, and reads with more than 20% of bases below Q15. Reads were mapped to GCF_015227675.2_mRatBN7.2.v2201 using HISAT. Gene-set alignment and expression quantification used Bowtie2 v2.3.4.3 and RSEM v1.3.1, respectively. Mean clean yield was 6.59 Gb per sample (range, 6.05–6.64); Q30 ranged from 88.75% to 91.09%; and mean genome-mapping rate was 98.19% (range, 97.78–98.46%). Sample identifiers and quality metrics are provided in Supplementary Tables 3 and 9. The expression matrix comprises 19,545 genes and 36 sample-specific read-count estimate columns, including fractional RSEM estimates. Differential-expression comparisons were performed separately within each sex using the BGI DEGseq workflow. Figure 6a and Supplementary Figure 18 define DEGs at *q <* 0.05 and —log_2_ fold change| ≥ 1. Figure 6c uses hypergeometric GO/KEGG enrichment results based on the supplier’s default DEG list at *q* ≤ 0.001 and —log_2_ fold change| ≥ 1, with enrichment *q <* 0.05. These different gene-list thresholds are specified separately. Figure 6b includes female Model-versus-Control DEGs with finite fold changes in both comparisons; seven non-finite pairs were excluded from the scatter plot only. The female DEG union comprises 1,270 genes. Complete expression, differential-expression, and enrichment tables are provided in Supplementary Data 1, with figure-level gene lists and numerical summaries in Supplementary Table 14 and Supplementary Data 3. These WT-only, within-sex analyses are exploratory and do not include a formal between-sex or genotype-by-treatment transcriptomic comparison. No additional batch-adjusted analysis was performed.

### 2.10 Statistical analysis

Animal-level values for 19 endpoints were analyzed using a full 2 × 2 × 3 factorial linear model with sex (female or male), genotype (WT or α7-KO) and treatment (Control, Model or Nicotine) as categorical factors, including all two-way and three-way interactions. The rat was the independent experimental unit; each observation represented one rat, with microscopic fields treated as subsamples. The primary treatment contrasts were equal-weight averages of the female and male model-estimated cell means, so sex was retained in the model without female-versus-male pairwise comparisons. Tests used the unscaled animal values.

Five fixed contrasts were evaluated for every endpoint: Model minus Control and Nicotine minus Model separately in WT and KO rats, plus the WT-minus-KO difference in the Nicotine–Model response. These five comparisons form one local Šídák family, with adjusted *P* = 1 − (1 − nominal P)^5^. The same family was used for all 19 endpoints, without choosing comparisons by observed significance. Different endpoints were not pooled into one study-wide correction; the comparison families were specified during revision and the analyses are exploratory. Complete estimates, standard errors, *t* statistics, degrees of freedom, nominal and adjusted *P-*values, and pointwise confidence intervals are provided in Supplementary Table P1.

For direct genotype-response comparisons, the Nicotine–Model response in KO rats was subtracted from that in WT rats after equal averaging across females and males. A significant response in one genotype and a nonsignificant response in the other was not treated as evidence that the responses differed. Sex-related response heterogeneity was evaluated using the sex × treatment and sex × genotype × treatment terms; their two *P*-values were Šídák-adjusted within each endpoint (Supplementary Table P2). No female-versus-male pairwise comparison or claim of a generalized sex difference was made.

Welch–Satterthwaite contrasts provided an unequal-variance sensitivity analysis for the same five sex-averaged contrasts, with a separate Šídák five-comparison family within each endpoint. Conclusions are qualified when pooled-variance and Welch results disagree. For display, each observation in Figures 2, 3, 4 and Supplementary Figures 2–4 was first divided by the mean of the same-sex WT Control group for that endpoint and was then pooled across sexes. Thus, the combined WT Control mean equals 1 and KO values use the same-sex WT denominator; individual observations retain variability. Circles denote females and squares denote males. This normalization affects only display: all models use original units. TUNEL remains in percentage units because male WT Control is zero. All observations for each endpoint were analyzed without imputation or pairing across endpoints.

Tissue SA-β-gal-positive area was analyzed separately using a sex × treatment linear model with six animal-level observations per treatment and sex. The sex-averaged Model–Control and Nicotine–Model contrasts were Šídák-adjusted across two comparisons (Supplementary Table P5). Figure 1B shows mean ± SD after same-sex Control normalization and pooling, with circles for females and squares for males. Tests use unscaled area measurements. This exploratory area analysis does not apply to the representative-field cell-body counts or culture images.

**FIGURE 1:**
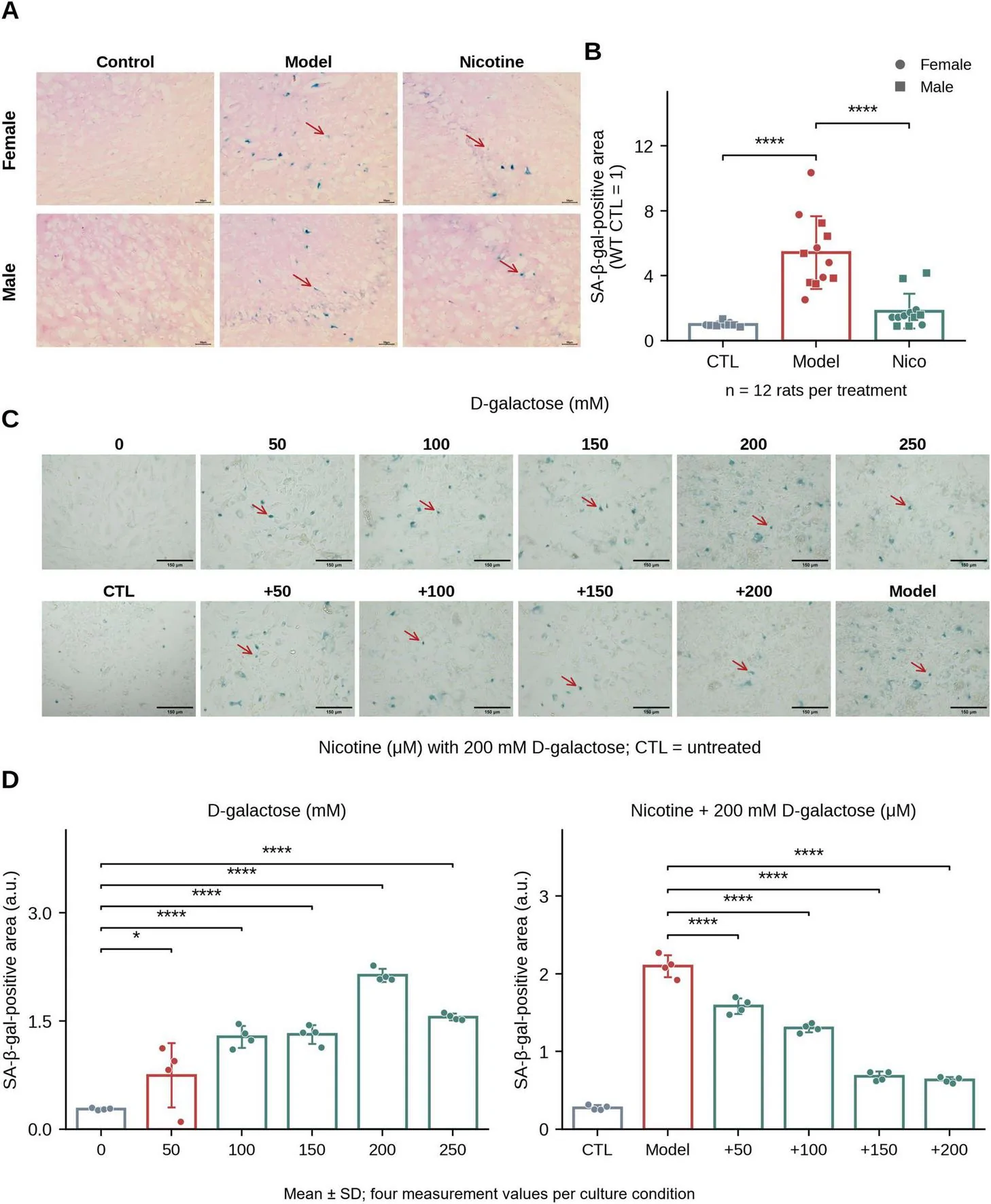
SA-β-gal staining in hippocampal tissue and primary glial cultures. **(A)** Representative tissue images from female and male wild-type rats in Control, Model, and Nicotine groups. **(B)** Hippocampal SA-β-gal-positive area, normalized to the same-sex Control mean for display; *n* = 12 rats per treatment, comprising six females and six males. A sex × treatment model was followed by sex-averaged Model–Control and Nicotine–Model contrasts with Šídák adjustment across two comparisons. **(C)** Representative culture images. The 200-mM D-galactose Model field is shared between the D-galactose and nicotine series. **(D)** Culture SA-β-gal-positive area; each point represents one well derived from one independent donor mouse, with four donor-derived wells per condition. Separate one-way ANOVAs were followed by Dunnett comparisons against 0 mM D-galactose or the D-galactose Model condition. Bars show mean ± SD. In the tissue plot, circles denote females and squares denote males. Arrows identify selected SA-β-gal-positive structures. Scale bars: 50 μm for tissue and 150 μm for culture. *P_*adj*_ < 0.05; **P_*adj*_ < 0.01; ***P_*adj*_ < 0.001; ****P_*adj*_ < 0.0001; ns, *P*_*adj*_ ≥ 0.05. Exact results are in Supplementary Tables P5 and S13.

For qPCR, all three treatment groups within each gene, sex and genotype used a common Control calibrator. Positive relative-expression values were log_2_-transformed and analyzed using sex-adjusted factorial models. Model–Control and Nicotine–Model contrasts were averaged equally across sexes and Šídák-adjusted across the four WT/KO treatment contrasts within each gene (Supplementary Table P4). Figure 5 and Supplementary Figure 5 show arithmetic means ± SD on the original relative-expression scale, with circles for females and squares for males. The WT and KO expression levels use separate Control calibrators. Inference was restricted to treatment contrasts within genotype; baseline expression levels were not compared between genotypes.

Culture SA-β-gal concentration-response datasets were analyzed separately using one-way ANOVA followed by two-sided Dunnett comparisons. Nonzero D-galactose concentrations were compared with 0 mM, and nicotine concentrations during 200 mM D-galactose exposure were compared with the Model condition. Each condition contained four independent donor-derived wells, with one well and one plotted value per donor mouse. Exact comparisons are provided in Supplementary Table 13 and Supplementary Data 2. Representative-field SA-β-gal cell-body counts in Supplementary Table 10 were treated descriptively and were not subjected to group-level significance testing. RNA-seq uses DEGseq and enrichment *q*-values as described in Section 2.9. The comparison families were specified during revision. Behavioral and hippocampal outcomes are distinguished from exploratory peripheral measures, and no study-wide multiplicity adjustment was applied across all endpoints. Full factorial-model tests are provided in Supplementary Table P6, and combined-sex sample sizes, means, and SDs underlying the plots are provided in Supplementary Table P7.

## 3 Results

### 3.1 SA-β-gal staining in hippocampal tissue and glial cultures

To assess whether the exposure produced a senescence-associated staining phenotype and whether nicotine co-treatment altered it, we quantified hippocampal SA-β-gal-positive area. Representative hippocampal staining is shown in Figure 1A. Tissue SA-β-gal-positive area was higher in Model than Control rats (sex-adjusted Šídák *P* = 5.00 × 10^–8^) and lower in Nicotine than Model rats (*P* = 2.44 × 10^–6^; Figure 1B and Supplementary Table P5). Display values were normalized to same-sex WT Control before pooling. Descriptive counts from representative fields are provided separately in Supplementary Table 10.

To examine the staining response in a complementary cell-culture system, we evaluated D-galactose concentration responses and nicotine co-exposure in astrocyte-enriched primary glial cultures (Figure 1C). In culture, SA-β-gal-positive area was higher than the 0-mM condition at 50 mM D-galactose (Dunnett-adjusted *P* = 0.0187) and at 100–250 mM (all adjusted *P* < 0.00001). During 200 mM D-galactose exposure, all tested nicotine concentrations (50–200 μM) were lower than Model (all adjusted *P* < 0.000001; Figure 1D and Supplementary Table 13). These exploratory culture results describe staining changes rather than cell viability or irreversible senescence.

### 3.2 Endpoint-specific water-maze responses

To determine whether nicotine co-treatment was associated with altered spatial performance, we examined water-maze outcomes and compared treatment responses directly between genotypes. In the sex-averaged analysis, WT Nicotine rats had lower latency to first target entry than WT Model rats (Šídák-adjusted *P* = 0.00527; Welch-adjusted *P* = 0.0182), lower mean distance to target (*P* = 0.000112; Welch *P* = 0.0267), and more platform-site crossings (*P* = 0.000114; Welch *P* = 0.00384; Figure 2 and Supplementary Table P1). None of the corresponding nicotine-versus-model comparisons in knockout rats was significant. The direct WT–KO nicotine-response difference was supported for platform-site crossings (*P* = 0.0159; Welch *P* = 0.00343), but not for latency or mean distance. Thus, the behavioral data support an endpoint-specific genotype-associated difference in the crossing response, rather than a uniform genotype difference across all behavioral measures. The latency endpoint refers to first target entry, not the acquisition trajectory over 5 days.

**FIGURE 2:**
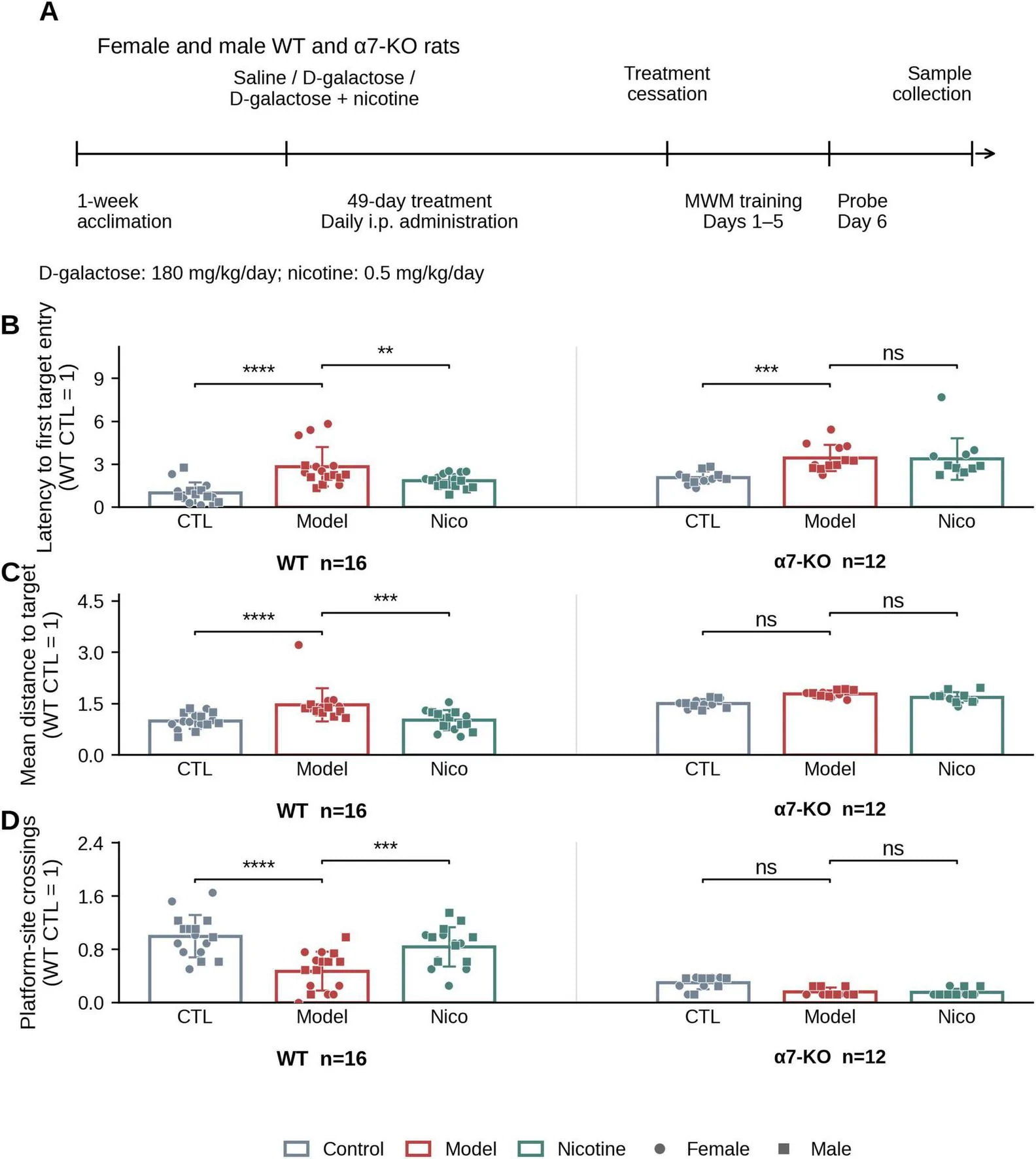
Experimental design and water-maze outcomes. **(A)** Treatment and testing schedule; training began approximately 24 h after the final administration. **(B)** Latency to first target entry. **(C)** Mean distance to target. **(D)** Platform-site crossings. Display values were divided by the same-sex WT Control mean; analyses used unscaled animal-level values. Each treatment included *n* = 16 WT rats and *n* = 12 α7-KO rats. Bars show mean ± SD; circles denote females and squares denote males. A sex × genotype × treatment model was followed by five sex-averaged contrasts per endpoint with Šídák adjustment. Brackets indicate within-genotype Model–Control or Nicotine–Model comparisons. *P_*adj*_ < 0.05; **P_*adj*_ < 0.01; ***P_*adj*_ < 0.001; ****P_*adj*_ < 0.0001; ns, *P*_*adj*_ ≥ 0.05. Direct WT–KO contrasts and Welch results are in Supplementary Table P1; plotting summaries are in Supplementary Table P7.

### 3.3 Hippocampal cytokine staining

To examine the hippocampal inflammatory component of the aging-like phenotype, we measured IL-1β, IL-6, and TNF-α immunoreactivity. Hippocampal cytokine staining is displayed after same-sex WT Control normalization and pooling (Figure 3). In WT rats, nicotine co-treatment was associated with lower IL-6 immunoreactivity than D-galactose alone (Šídák-adjusted *P* = 0.0159; Welch *P* = 0.0061) and lower TNF-α immunoreactivity (*P* = 0.00982; Welch *P* = 0.00597). IL-1β did not meet the pooled-variance threshold (adjusted *P* = 0.120), although the Welch sensitivity result was just below 0.05 (*P* = 0.0485). The nicotine-versus-model comparison for TNF-α in knockout rats met the pooled-model threshold (*P* = 0.00465) but not the Welch threshold (*P* = 0.167). No direct WT–KO nicotine-response contrast for these three cytokines met the adjusted threshold. Hippocampal IL-6 showed a significant sex × genotype × treatment term after the two-test within-endpoint adjustment (*P* = 0.0286); the sex-averaged effect should therefore not be interpreted as uniform in females and males.

**FIGURE 3:**
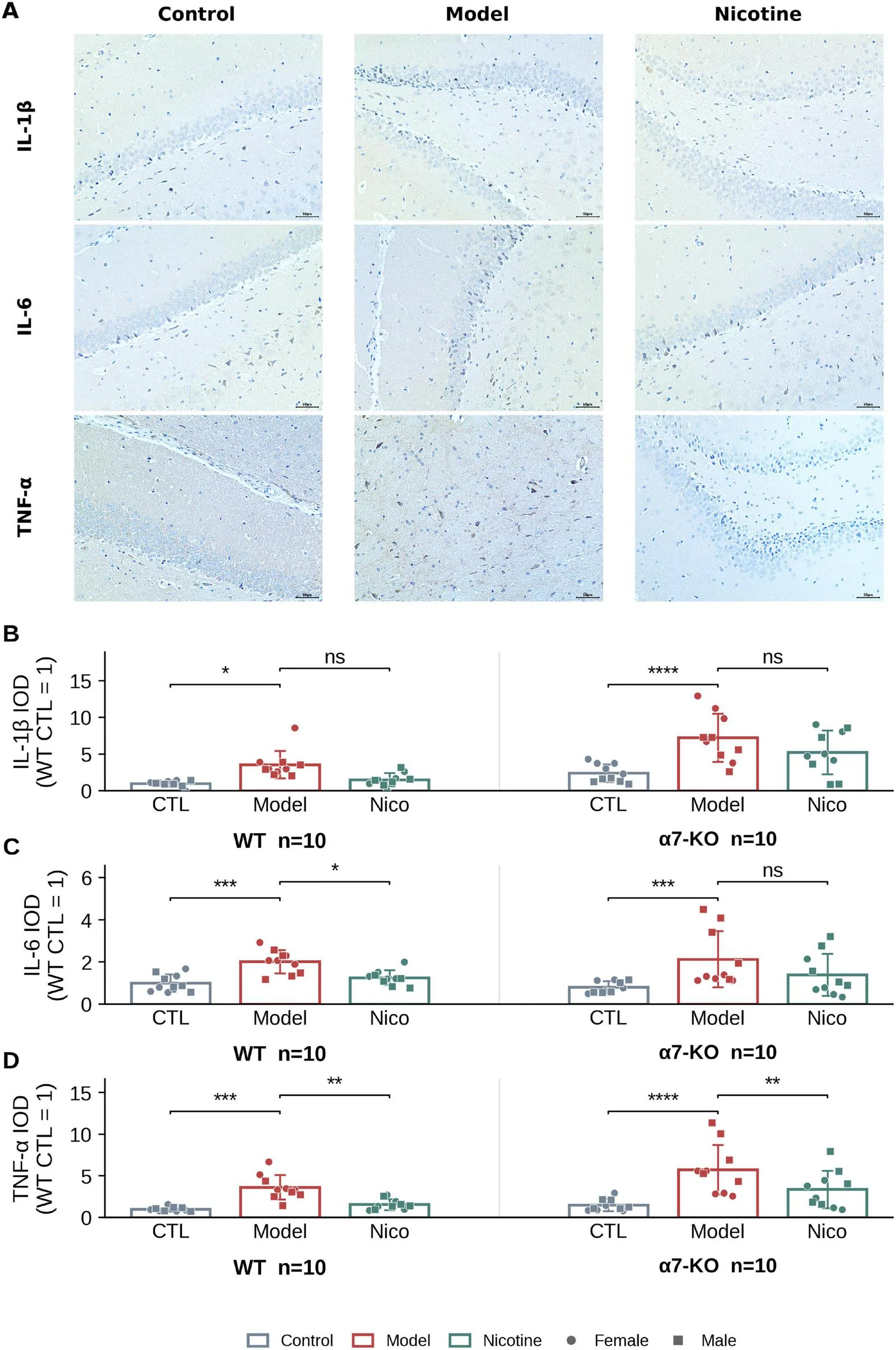
Hippocampal cytokine immunoreactivity. **(A)** Representative IL-1β, IL-6, and TNF-α staining in female WT rats; corresponding fields are included in Supplementary Figure 7. Scale bars, 50 μm. **(B–D)** IL-1β, IL-6, and TNF-α integrated optical density, normalized to the same-sex WT Control mean for display; *n* = 10 rats per genotype and treatment, comprising five females and five males. Bars show mean ± SD; circles denote females and squares denote males. Unscaled animal-level measurements were analyzed using sex × genotype × treatment models with five Šídák-adjusted contrasts per endpoint. Brackets indicate within-genotype Model–Control or Nicotine–Model comparisons. Hippocampal IL-6 showed sex × genotype × treatment heterogeneity (Supplementary Table P2). *P_*adj*_ < 0.05; **P_*adj*_ < 0.01; ***P_*adj*_ < 0.001; ****P_*adj*_ < 0.0001; ns, *P*_*adj*_ ≥ 0.05. Exact results and plotting summaries are in Supplementary Tables P1 and P7.

### 3.4 Glial immunoreactivity and serum GSH

To assess whether the hippocampal response involved glial changes, we quantified GFAP and IBA1 immunoreactivity as astrocytic and microglial readouts (Figure 4A). After sex averaging, WT Nicotine rats had lower GFAP (Šídák-adjusted *P* = 0.00987; Welch *P* = 0.0128) and IBA1 immunoreactivity (*P* = 3.06 × 10^–6^; Welch *P* = 0.00582) than WT Model rats (Figures 4B, C). The direct WT-minus-KO nicotine-response difference for IBA1 was significant under both pooled-variance and Welch analyses (adjusted *P* = 0.0149 and 0.0464), whereas the corresponding GFAP difference was not.

**FIGURE 4:**
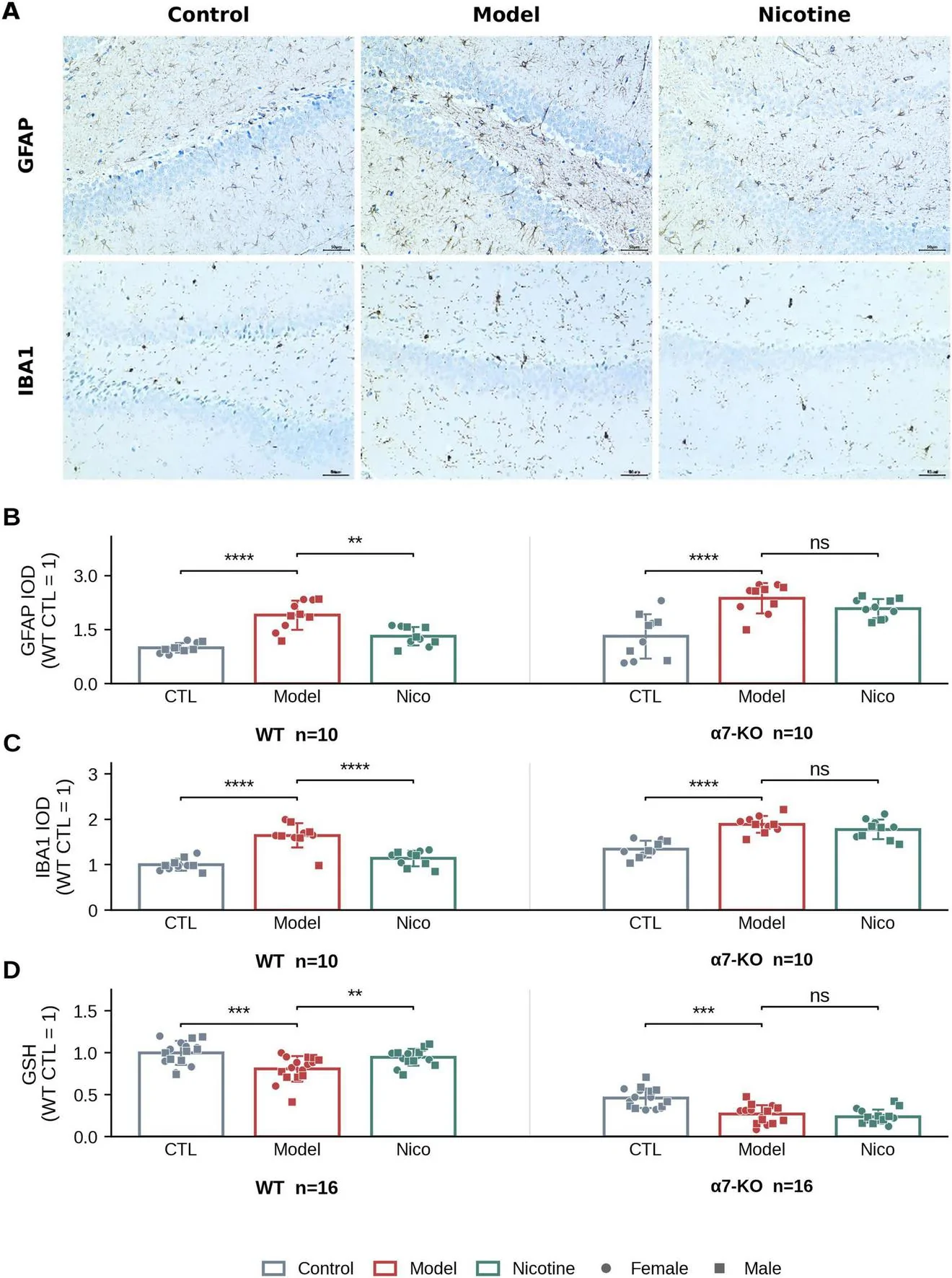
Hippocampal glial immunoreactivity and serum GSH. **(A)** Representative GFAP and IBA1 staining in female WT rats. Scale bars, 50 μm. **(B, C)** GFAP and IBA1 integrated optical density. **(D)** Serum GSH concentration. Display values were normalized to the same-sex WT Control mean. GFAP and IBA1 analyses included *n* = 10 rats per genotype and treatment, with five per sex; GSH included n = 16, with eight per sex. Bars show mean ± SD; circles denote females and squares denote males. Unscaled measurements were analyzed using sex × genotype × treatment models with five Šídák-adjusted contrasts per endpoint. Brackets indicate within-genotype comparisons. *P_*adj*_ < 0.05; **P_*adj*_ < 0.01; ***P_*adj*_ < 0.001; ****P_*adj*_ < 0.0001; ns, *P*_*adj*_ ≥ 0.05. Direct WT–KO contrasts and Welch results are in Supplementary Table P1; plotting summaries are in Supplementary Table P7.

To assess the accompanying systemic antioxidant response, we measured serum GSH separately from the hippocampal staining outcomes. Serum GSH was lower in WT Model than WT Control rats (*P* = 0.000131; Welch *P* = 0.00641) and higher in WT Nicotine than WT Model rats (*P* = 0.00927; Welch *P* = 0.0313; Figure 4D). The direct WT-minus-KO nicotine-response difference for GSH was also supported under both analyses (*P* = 0.0336; Welch *P* = 0.0262). These results support endpoint-specific genotype-associated responses for IBA1 immunoreactivity and serum GSH, but do not establish a generalized α7-dependent mechanism. These measurements do not establish whether hippocampal GSH changed.

### 3.5 Hippocampal transcripts related to APP processing

To explore whether the aging-like phenotype was accompanied by transcriptional changes in APP-processing genes, we measured hippocampal *App*, *Bace1*, and *Psen1* mRNA. These genes encode the precursor substrate, the β-secretase, and a component of γ-secretase, respectively (Vassar et al., 1999; De Strooper et al., 1998). D-galactose-treated WT rats showed higher *App*, *Bace1*, and *Psen1* relative mRNA expression than WT controls (Figure 5 and Supplementary Table P4). In the sex-adjusted qPCR analysis, WT Nicotine rats had lower *App*, *Bace1* and *Psen1* mRNA levels than WT Model rats (Šídák-adjusted *P* = 0.00743, 2.56 × 10^–5^ and 0.00227, respectively; Figure 5 and Supplementary Table P4). *Ache* was also lower in WT Nicotine than WT Model rats (*P* = 0.0149; Supplementary Figure 5). None of the corresponding nicotine-versus-model comparisons in knockout rats met the adjusted threshold. WT and KO values use separate Control calibrators; the qPCR analysis reports treatment contrasts within genotype. These transcript measurements do not determine protein abundance, secretase activity, or Aβ production.

**FIGURE 5:**
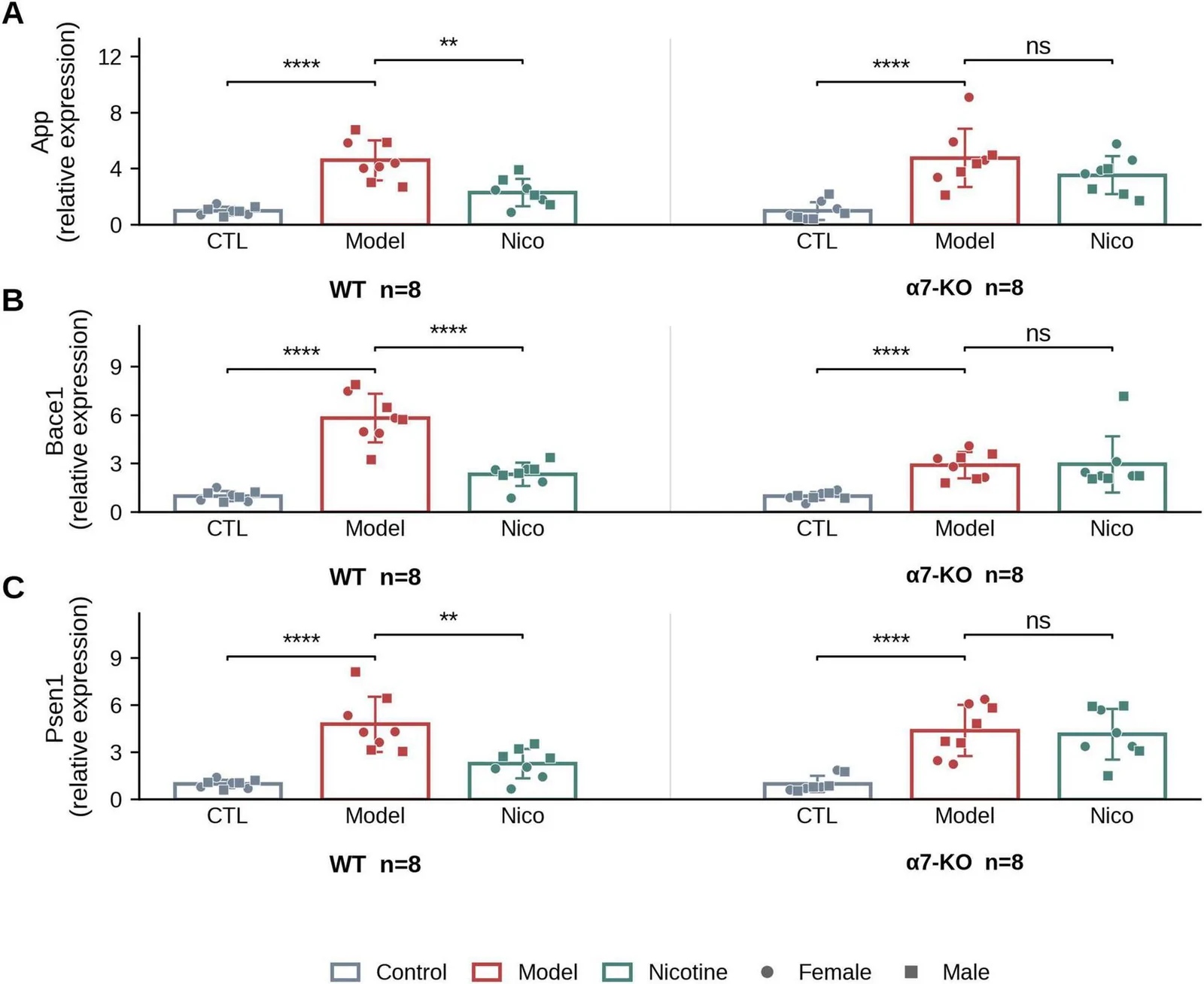
Hippocampal qRT-PCR results. Relative mRNA expression of **(A)** *App*, **(B)** *Bace1*, and **(C)** *Psen1* is shown by genotype, with female and male observations displayed together; *n* = 8 rats per genotype and treatment, comprising four per sex. Bars show mean ± SD; circles denote females and squares denote males. Values were calibrated to the common Control within each gene, sex, and genotype. Sex-adjusted models on log_2_ (relative expression) were followed by four sex-averaged Model–Control and Nicotine–Model contrasts per gene, with Šídák adjustment. *P_*adj*_ < 0.05; **P_*adj*_ < 0.01; ***P_*adj*_ < 0.001; ****P_*adj*_ < 0.0001; ns, *P*_*adj*_ ≥ 0.05. These analyses concern within-genotype treatment responses; direct genotype-response contrasts were not performed. *Ache* is shown in Supplementary Figure 5.

### 3.6 Exploratory hippocampal transcriptomic profiles in wild-type rats

To place the targeted behavioral and tissue findings in a broader molecular context, we performed exploratory RNA sequencing of wild-type hippocampi. Using a threshold of *q <* 0.05 and —log_2_ fold-change| ≥ 1, female hippocampal tissue showed 874 differentially expressed genes in the Model-versus-Control comparison and 684 in the Nicotine-versus-Model comparison, with 288 genes shared between the two comparisons (union, 1,270 genes; Figure 6). In male hippocampal tissue, the same threshold yielded 213 differentially expressed genes in the Model-versus-Control comparison and 400 in the Nicotine-versus-Model comparison. These within-sex counts are not a formal test of a sex difference.

**FIGURE 6:**
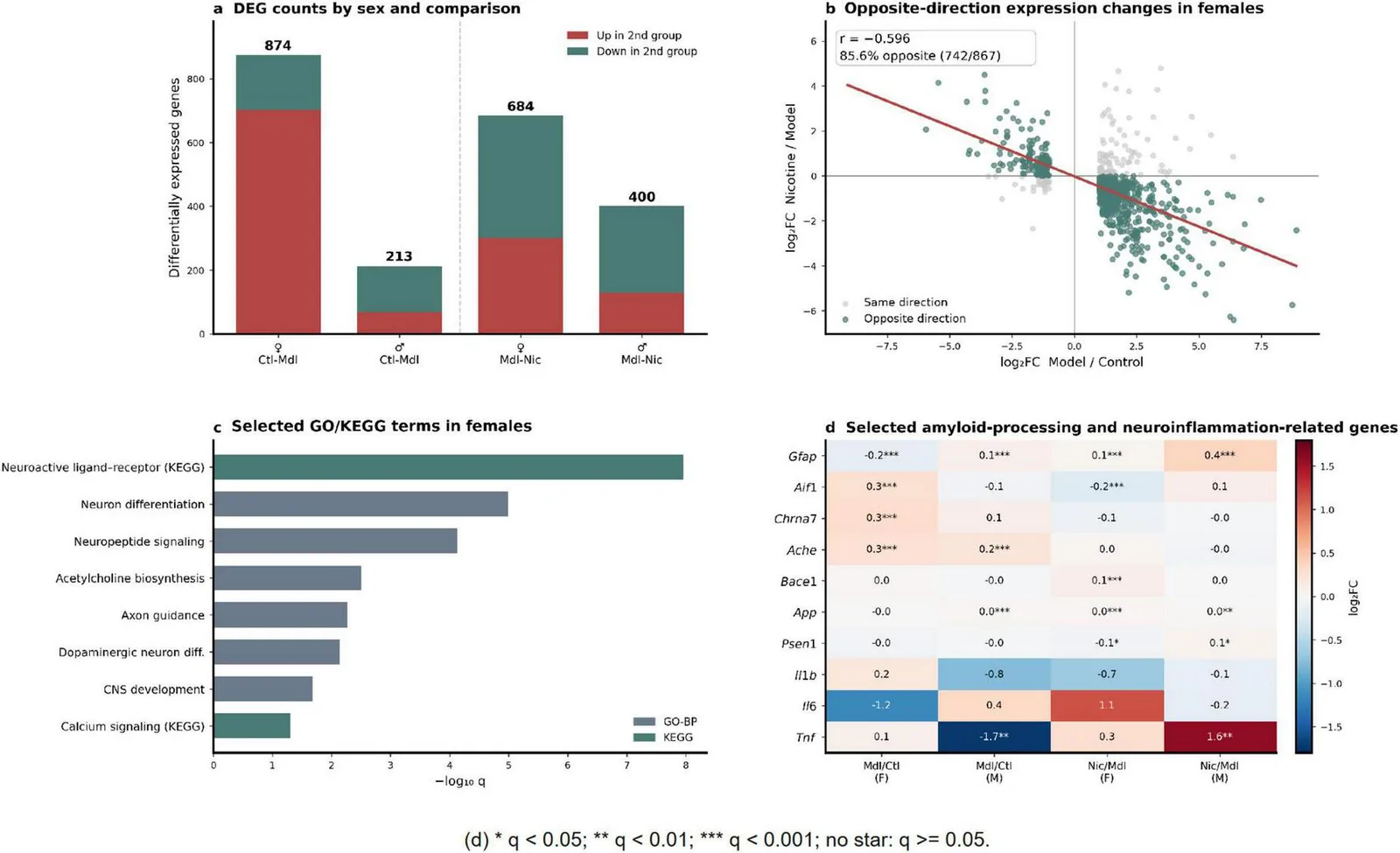
Hippocampal transcriptomic responses in wild-type rats. RNA-seq included six independent rats per sex and treatment, yielding 36 samples. Nicotine denotes D-galactose plus nicotine. **(a)** Within-sex DEG counts at *q <* 0.05 and — log_2_ fold change — ≥ 1. Increased and decreased expression refer to Model relative to Control or Nicotine relative to Model. **(b)** Fold-change relationship for 867 of the 874 female Model-versus-Control DEGs with finite values in both comparisons. Pearson r = −0.596; 742/867 genes showed opposite-direction changes. Seven non-finite pairs were excluded from this panel only. The comparisons share the Model group, and the association is descriptive. **(c)** Selected neural GO biological-process and KEGG terms for female Model versus Control, using hypergeometric enrichment results based on the supplier’s default DEG list at *q* ≤ 0.001 and — log_2_ fold change — ≥1. Bars show −log_10_ enrichment q values. **(d)** Selected-gene log_2_ fold changes. Stars denote DEGseq q values: *q < 0.05; **q < 0.01; ***q < 0.001; no star, q ≥ 0.05. Stars indicate the q-value criterion only; a value rounded to 0.0 may retain a star. The female DEG-union heatmap is in Supplementary Figure 18. Gene lists and numerical summaries are provided in Supplementary Table 14 and Supplementary Data 1 and 3.

The supplier GO and KEGG results for the female Model-versus-Control signature showed enrichment of neuronal differentiation, neuropeptide signaling, acetylcholine biosynthesis, axon guidance, neuroactive ligand-receptor interaction and calcium signaling (Figure 6c). Among the 874 female Model-versus-Control DEGs, 867 had finite log_2_ fold changes in both comparisons. For these 867 genes, the Model-versus-Control and Nicotine-versus-Model vectors were negatively correlated (r = −0.596, rounded to −0.60), and 742 genes showed opposite-direction changes (85.6%, rounded to 86%; Figure 6b). These statistics describe the Model-versus-Control DEG set, not the 288-gene intersection. Because both contrasts share the Model group, this descriptive relationship does not establish independent molecular rescue. The transcriptomic dataset contains wild-type rats only and therefore does not provide genotype-resolved evidence of an α7-nAChR-dependent mechanism. Supplementary Figure 18 displays the 1,270-gene female union across 18 samples, with expression patterns differing between groups.

### 3.7 Exploratory apoptosis, oxidative, systemic inflammatory, and peripheral biomarker outcomes

To examine whether the hippocampal response extended to DNA fragmentation, we assessed TUNEL-positive nuclei. TUNEL-positive nuclei were lower in Nicotine than Model rats in WT and KO groups after sex averaging (Šídák-adjusted *P* = 1.01 × 10^–5^ and 3.69 × 10^–7^; Welch *P* = 0.0128 and 0.00247; Supplementary Figure 6). The direct WT–KO nicotine-response contrast was not significant. These staining observations do not establish that labeled nuclei were neuron-specific or that a common receptor-dependent mechanism explains the changes.

To complement serum GSH with additional peripheral redox measures, we quantified serum SOD concentration and MDA. Serum SOD and MDA are displayed after same-sex WT Control normalization and pooling (Supplementary Figure 2). Nicotine-co-treated WT rats had higher SOD concentration than WT Model rats (Šídák-adjusted *P* = 0.0226; Welch *P* = 0.0107) and lower MDA (*P* = 0.0183), although the MDA comparison did not remain significant under Welch sensitivity analysis (*P* = 0.0546). The serum GSH results are presented in Figure 4D. The direct WT–KO nicotine-response differences for SOD and MDA were not significant.

To compare the tissue findings with systemic inflammatory responses, we measured serum IL-1β, IL-6, and TNF-α. In WT rats, serum IL-6 and TNF-α were lower with nicotine co-treatment than with D-galactose alone (Šídák-adjusted *P* = 4.72 × 10^–5^ and 0.000457; Welch *P* = 0.00476 and 0.0145; Supplementary Figure 3). The nicotine-versus-model comparison for IL-1β in knockout rats met the pooled-model threshold (*P* = 0.0202) but not the Welch threshold (*P* = 0.158). The direct WT–KO nicotine-response difference for serum IL-6 met the pooled-model threshold (*P* = 0.0432) but not Welch sensitivity (*P* = 0.0627). These findings do not establish a generalized α7-dependent systemic cytokine effect.

To characterize additional systemic changes without assuming that they reflect hippocampal pathology, we examined serum ApoE, total Tau, and Aβ1–42 and urinary formate as exploratory outcomes. In WT rats, ApoE and Tau were lower with nicotine co-treatment than with D-galactose alone (Supplementary Figure 4) under both variance assumptions (ApoE *P* = 2.02 × 10^–6^, Welch *P* = 0.00659; Tau *P* = 0.00254, Welch *P* = 0.00667). The nicotine-versus-model comparison for urinary formate in WT rats met the pooled threshold (*P* = 5.76 × 10^–5^) but not Welch sensitivity (*P* = 0.0623); the corresponding comparison in knockout rats met both thresholds (*P* = 0.0424; Welch *P* = 0.000204). Serum Aβ1–42 showed no significant treatment contrast, and none of the direct genotype-response contrasts for these peripheral measures was significant. Peripheral measurements alone do not establish hippocampal amyloid pathology or its reversal (Belloy et al., 2019).

## 4 Discussion

Nicotine co-treatment was associated with improvements in selected behavioral and hippocampal outcomes during D-galactose exposure. Wild-type rats receiving nicotine had better performance on the three reported water-maze measures, higher serum GSH, and lower hippocampal GFAP and IBA1 immunoreactivity. Direct genotype comparisons supported differences in the nicotine response for platform-site crossings, serum GSH, and hippocampal IBA1 under both pooled-variance and unequal-variance analyses. These findings support an α7-nAChR-associated contribution to specific responses, rather than a single mechanism that explains every measured outcome.

The nicotine dose has experimental precedent, but exposure depends on route, duration, and species. Majdi et al. (2018) tested subcutaneous nicotine at 0.1, 0.5, and 1 mg/kg in D-galactose-treated mice; 0.5 mg/kg was among the doses associated with behavioral improvement. This supports the dose magnitude used here, not equivalent exposure in rats receiving intraperitoneal injections. The metabolic effects reported by Yang et al. (2023) and Jia et al. (2025) further illustrate the range of possible nicotine responses. None of these studies establishes a common safety threshold or a clinical recommendation.

The water-maze findings indicate an association between nicotine co-treatment and selected measures of spatial performance. The direct genotype difference was supported for platform-site crossings, but not for latency to first target entry or mean distance to the target. We therefore do not infer a uniform α7-dependent effect across behavioral endpoints. Training began approximately 24 h after the final administration. Kenney et al. (2011) showed that nicotine withdrawal can alter spatial object recognition in mice under a different exposure regimen. Our design cannot separate persistent treatment effects from withdrawal-related influences, and the reported endpoint analysis is distinct from a repeated-measures analysis of learning across the five training days.

The glial findings provide a tissue-level counterpart to the behavioral response. Nicotine co-treatment reduced GFAP and IBA1 immunoreactivity in wild-type hippocampi, but only IBA1 showed a direct genotype-response difference supported by both variance analyses. Immunoreactivity describes the staining phenotype; it does not, by itself, establish a particular glial functional state. Prior studies of nicotine and α7-selective ligands support the plausibility of altered glial inflammatory signaling, while also showing that the response depends on the experimental context (Park et al., 2022; Cao et al., 2024; Lombardi et al., 2025).

Serum GSH was examined as a peripheral component of the antioxidant response. Its increase with nicotine co-treatment and the direct genotype-response difference are compatible with α7-nAChR-associated modulation of systemic redox regulation. They do not demonstrate restoration of brain glutathione. Brain GSH was not measured, and no serum–hippocampal GSH relationship was tested. The serum result therefore cannot identify the tissue responsible for the change or establish that glutathione mediates the behavioral or hippocampal effects.

One possible explanation is that α7-associated anti-inflammatory and antioxidant signaling reduces oxidative demand and/or supports glutathione synthesis and recycling. This is a mechanistic hypothesis, not a pathway demonstrated here. In astrocytes, Patel et al. (2017) linked α7 stimulation to suppression of NF-κB signaling and Nrf2-dependent antioxidant responses. Importantly, increased Gclc transcript expression in that study was not accompanied by a detectable increase in GCLC protein, illustrating why transcript changes alone are insufficient. Shih et al. (2003) demonstrated that Nrf2 activation can coordinate glial glutathione synthesis and release, while Zhang et al. (2023) implicated α7-nAChR/Nrf2/HO-1 signaling in a D-galactose model treated with PNU282987. These separate experiments support biological plausibility but do not establish the source of the serum GSH response to nicotine in our rats. We did not measure brain GSH, oxidized glutathione, glutathione-synthetic or recycling enzymes, or Nrf2/HO-1 activation. Consequently, reduced oxidative consumption, altered synthesis, and altered recycling cannot be distinguished in the present dataset.

The genotype inference rests on direct comparisons of treatment responses, not on significance in wild-type rats and nonsignificance in knockout rats. This distinction is essential because the latter pattern alone does not show that the effects differ. Both cohorts had a Sprague–Dawley background, but they came from different sources. Developmental compensation and cohort-associated differences could therefore contribute to the observed responses. Genotyping establishes the targeted allele, not hippocampal receptor loss at the protein or functional level.

The other peripheral outcomes describe systemic responses rather than a direct hippocampal mechanism. Serum ApoE and total Tau were lower with nicotine co-treatment in wild-type rats, whereas serum Aβ1–42 did not change significantly. The urinary formate response was sensitive to the variance assumption in wild-type rats, and its direct genotype-response difference was not significant. Peripheral ApoE and formate measurements have distinct biological contexts and should not be treated as interchangeable measures of brain pathology (Belloy et al., 2019; Morrow et al., 2015).

SA-β-gal staining decreased with nicotine co-treatment in hippocampal tissue and astrocyte-enriched primary glial cultures. This finding is consistent with modulation of a senescence-associated staining phenotype, but does not establish irreversible cell-cycle arrest or its reversal. Published astrocyte studies provide concentration- and duration-specific context: Aryal et al. (2021) reported no decrease in the MTT readout after 24 h at 0.1–10 μM nicotine; Lombardi et al. (2025) investigated higher-concentration responses; Revathikumar et al. (2016) studied 1–100 μM during 20-h inflammatory stimulation; and Patel et al. (2017) used 200 μM in a brief receptor-binding competition assay. These different settings do not demonstrate viability during our 7-day combined exposure. The separate BV2 screening data in Supplementary Table 15 are likewise distinct from the primary-culture SA-β-gal experiment.

The App, Bace1, and Psen1 panel was included to explore APP-processing-related transcription, not to establish an amyloid-driven mechanism. APP is the precursor substrate, BACE1 mediates β-site cleavage, and presenilin-1 participates in γ-secretase-dependent processing (Vassar et al., 1999; De Strooper et al., 1998). Nicotine-co-treated wild-type rats had lower hippocampal levels of these transcripts than rats receiving D-galactose alone. Oxidative stress can alter BACE expression and activity in neuronal experimental systems (Tamagno et al., 2002), providing a rationale for examining the panel during a redox and inflammatory challenge. A possible interpretation is that nicotine-associated changes in cellular stress are accompanied by altered APP-processing-related gene expression. We did not test this causal sequence, and the transcript results are not evidence that nicotine directly inhibits either secretase.

The apparent discrepancy between *Bace1* expression and Aβ requires particular caution. *Bace1* mRNA abundance does not establish BACE1 protein abundance or catalytic activity. O’Connor et al. (2008) demonstrated stress-dependent translational regulation of BACE1, emphasizing that RNA and protein measurements are not interchangeable. In addition, our qPCR measurements were made in hippocampus, whereas the quantitative Aβ assay measured Aβ1–42 in serum. Human measurements of central-to-peripheral Aβ efflux illustrate why circulating Aβ cannot be interpreted solely as a readout of brain peptide production (Roberts et al., 2014). That study does not establish peptide handling in our rats, and serum Aβ1–42 is not a direct readout of hippocampal amyloidogenic processing. Differences in compartment, post-transcriptional regulation, and sampling time are plausible explanations for discordant readouts, but were not tested here. A nonsignificant serum Aβ1–42 comparison neither establishes equivalence between groups nor demonstrates the absence of a brain change.

Corresponding APP, BACE1, and presenilin-1 protein measurements were not included, so the qPCR panel cannot establish enhanced or suppressed amyloidogenic processing. We did not measure secretase activity, APP cleavage products, or matched quantitative hippocampal Aβ species. The expression findings are therefore interpreted as exploratory transcriptional changes rather than evidence of reduced amyloid generation or plaque clearance. Broader work on oxidative stress, glial cytokine signaling, and astrocyte-derived ApoE supports a biological connection with amyloid-related pathways (Kumari et al., 2023; Deng et al., 2024; Smith et al., 1996; Mahan et al., 2022; Preman et al., 2024), but does not substitute for these measurements in our study.

Sex was retained in the joint genotype-by-treatment models, and treatment contrasts averaged the female and male responses equally. This approach avoids defining a sex difference from separate significant and nonsignificant comparisons. Hippocampal IL-6 showed a significant adjusted sex × genotype × treatment term, so its averaged response should not be interpreted as uniform across sexes. We do not claim generally greater nicotine efficacy in either sex.

The wild-type hippocampal transcriptome provides exploratory pathway context. Enrichment of neuronal processes, neuroactive ligand–receptor interactions, and calcium signaling in the female model-versus-control comparison suggests candidate systems for further investigation. The opposite-direction expression pattern across the two treatment contrasts is descriptive because both contrasts share the Model group. Cheng and Yakel (2015) showed that α7-nAChR activation can increase neuronal cAMP through adenylyl cyclase, but this mechanism was not tested here. The RNA-seq dataset contains no knockout samples and cannot establish an α7-dependent transcriptional mechanism.

### 4.1 Study limitations

The D-galactose challenge models selected aging-associated processes, not the full course of natural aging or established Alzheimer’s disease. Wild-type and knockout rats shared a genetic background but came from different sources, and developmental compensation may contribute to genotype-associated responses. Hippocampal α7-nAChR protein loss and receptor function were not measured. The absence of a nicotine-only group prevents separation of an independent nicotine effect from its interaction with D-galactose. Behavioral testing after treatment cessation cannot distinguish persistent treatment effects from withdrawal-related influences, and the reported outcomes do not constitute a 5-day repeated-measures analysis.

The measurements also limit mechanistic interpretation. GSH was measured in serum, not brain, and no paired tissue glutathione measurements or pathway interventions establish a glutathione-mediated brain effect. *App*, *Bace1*, and *Psen1* were assessed as transcripts without corresponding protein, secretase-activity, or APP-cleavage-product measurements. Serum Aβ1–42 is not a substitute for matched quantitative hippocampal Aβ. Thus, neither a brain antioxidant pathway nor altered amyloidogenic processing can be assigned as the causal mediator of nicotine’s effects. The proposed Nrf2-linked explanation is based on other experimental studies, not on direct pathway validation in these rats.

Finally, SA-β-gal staining does not establish irreversible senescence or culture viability, and the representative-field cell-body counts are descriptive rather than independent animal-level counts. TUNEL labeling was not assigned to a specific cell type. Urinary formate was not corrected for urine concentration. Small group sizes, variance sensitivity in some comparisons, sex-related heterogeneity in hippocampal IL-6, and within-endpoint rather than study-wide multiplicity correction warrant an exploratory interpretation. The wild-type-only RNA-seq analysis supplies pathway hypotheses but does not test genotype-dependent transcriptional responses.

### 4.2 Conclusions

Nicotine co-treatment attenuated selected behavioral and hippocampal alterations during D-galactose exposure. Direct genotype-response differences in platform-site crossings, serum GSH, and hippocampal IBA1 support an α7-nAChR-associated contribution to these specific outcomes. The serum and transcript findings do not establish restoration of brain glutathione or suppression of amyloid production. The study therefore identifies specific treatment-response associations and testable mechanistic hypotheses, rather than a unified receptor-dependent pathway or a clinical benefit of nicotine.

## Data Availability

The RNA-seq data are available in the Genome Sequence Archive (GSA) under accession CRA050070 and BioProject PRJCA074229. Supplementary Data 1 contains expression, differential-expression, enrichment, and qPCR quality-control files. Supplementary Data 2 contains animal-level measurements and statistical analyses, including joint-model contrasts and plotting summaries. Supplementary Data 3 contains Figure 6 gene lists and numerical summaries. Further sample details, quality metrics, sensitivity analyses, and culture comparisons are provided in the Supplementary material.
