# Supplementary material for: Nicotine attenuates D-galactose- induced hippocampal aging-like alterations with α7-nAChR- associated mechanisms
Source: Front Aging Neurosci. 2026 Oct 19;18:1947643. doi: 10.3389/fnagi.2026.1947643 (PMC13724548; doi:10.3389/fnagi.2026.1947643)
Supplement: Supplementary file 1 [file Data_Sheet_1.pdf]

Supplementary Material

Nicotine Attenuates D-Galactose-Induced Hippocampal Aging-Like Alterations with α7-nAChR-Associated Mechanisms

Supplementary Figure S1. Genotype identification of α7-nAChR knockout rats

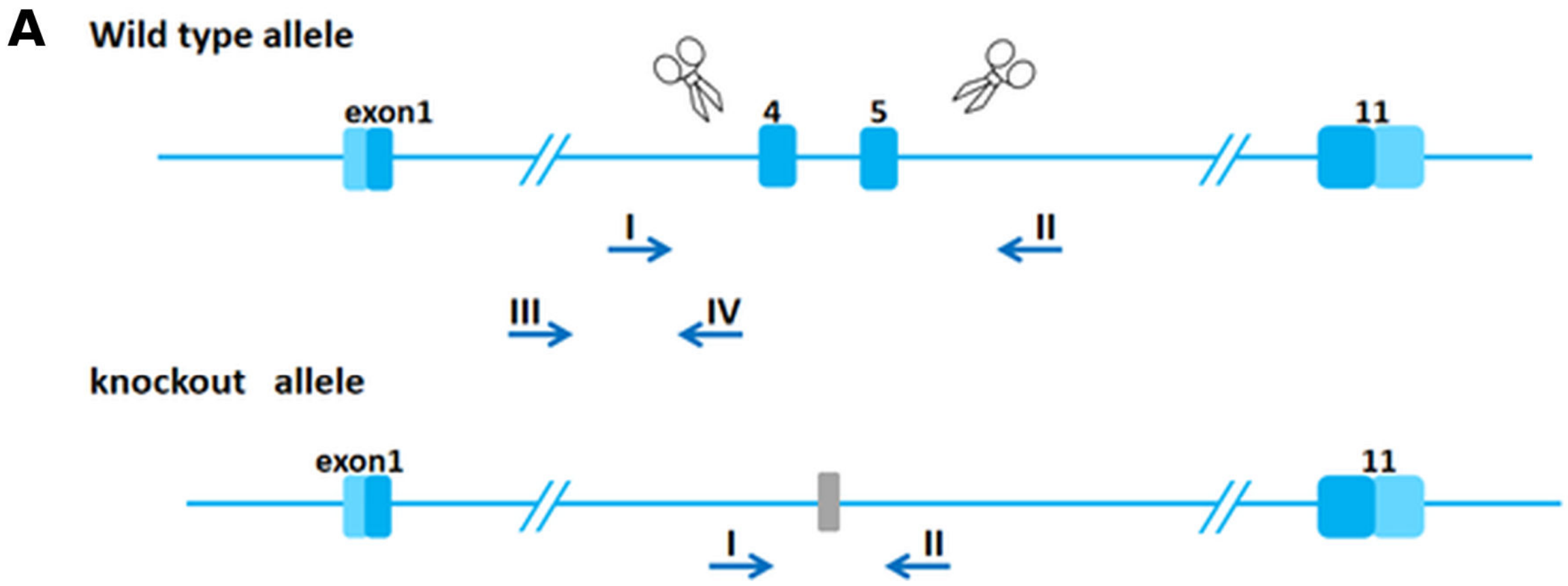

B

Primer	Sequence (5’-3’)	Primer type	Fragment length (bp)
P1	CAGGACTGTAGGCCT TTCCG	Forward	1756/601
P2	CAGCAGCAAACGAGA AGGTG	Reverse	
P3	AAGGGTGCCCGCAGA AGTT	Forward	284/0
P4	AATCGCATTTGGGGA CCTG	Reverse	

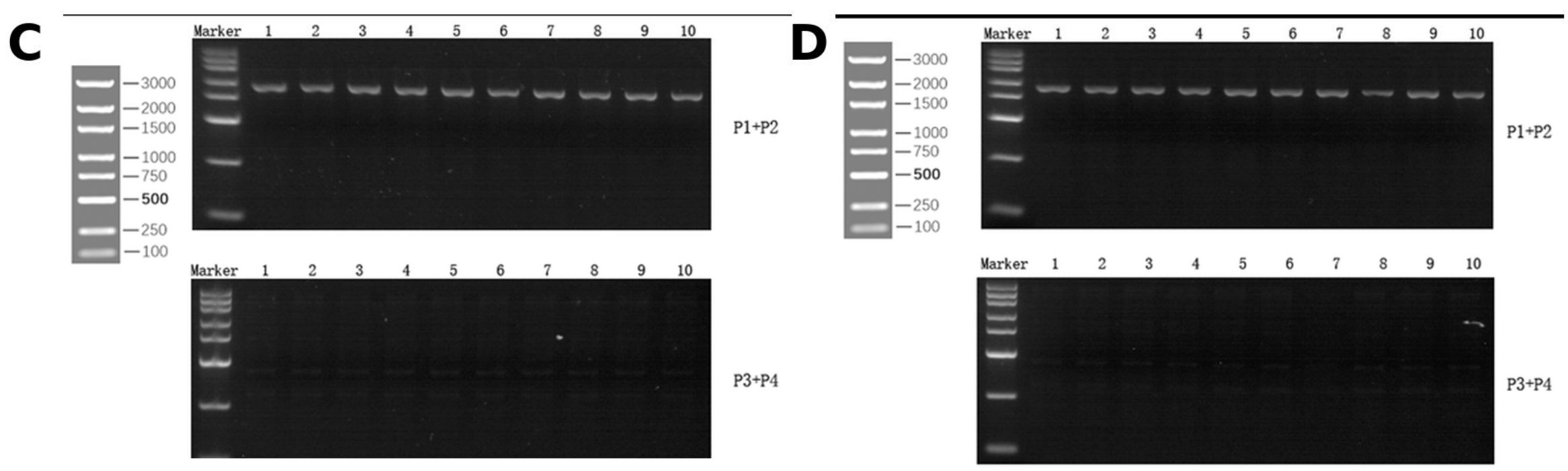

Supplementary Figure S1. Genomic targeting and PCR genotyping of α7-nAChR knockout rats. (A) CRISPR/Cas9 targeting of exons 4 and 5 of *Chrna7*-203 (ENSRNOT00000051883.5). (B) Primers, 5′–3′: P1, CAGGACTGTAGGCCTTTCCG; P2, CAGCAGCAAACGAGAAGGTG; P3, AAGGGTGCCCGCAGAAGTT; P4, AATCGCATTTGGGGACCTG. P1 and P3 are forward primers; P2 and P4 are reverse primers. Expected products are 1756 bp for WT and 601 bp for KO with P1/P2, and 284 bp for WT with no corresponding KO product with P3/P4. (C, D) Panels from separate sets of animals. Upper and lower gels show P1/P2 and P3/P4 reactions, respectively. These assays identify genomic allele status.

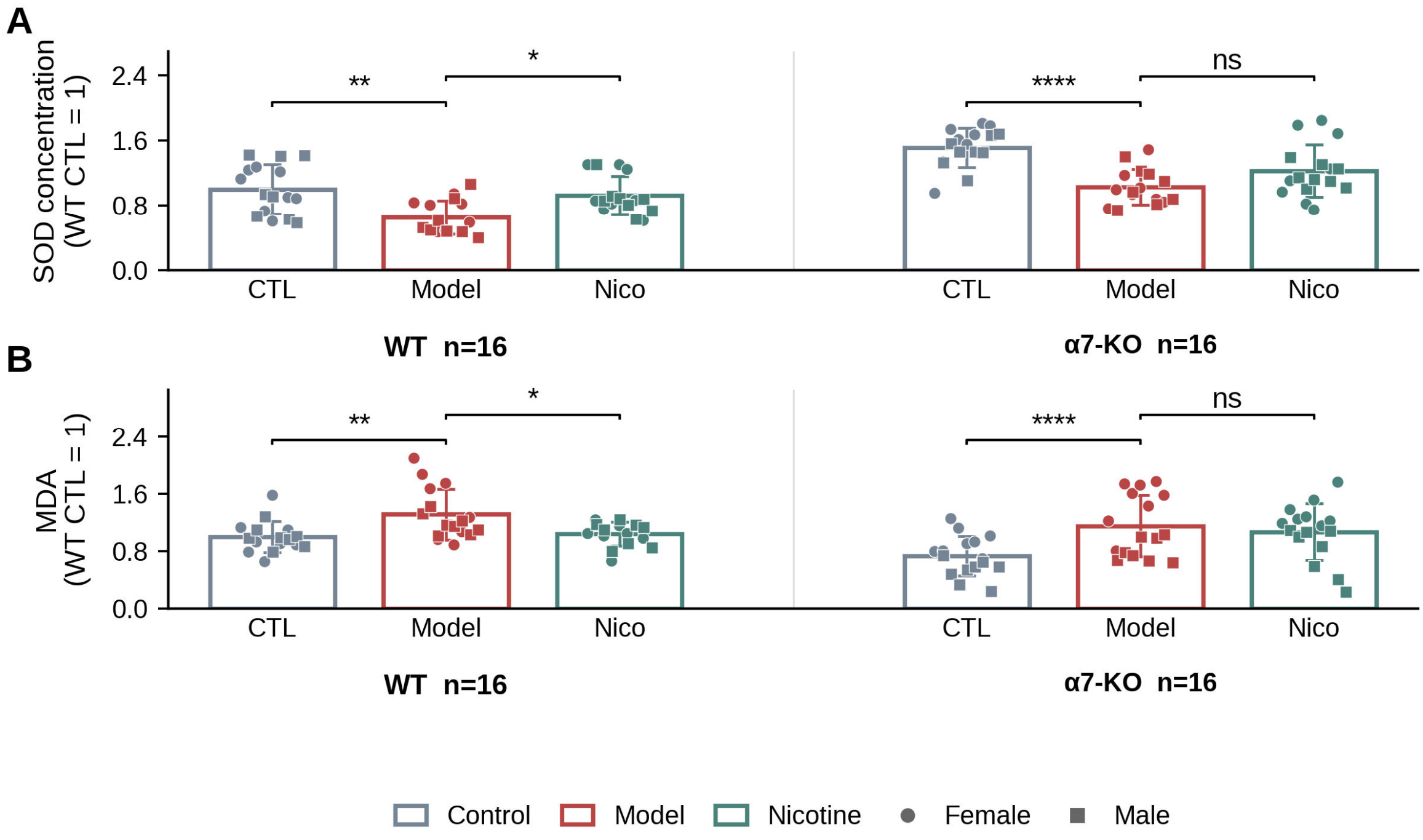

Supplementary Figure S2. Serum SOD concentration and MDA. Display values were normalized to the same-sex WT Control mean; n = 16 rats per genotype and treatment. Bars show mean ± SD; circles denote females and squares denote males. Unscaled measurements were analyzed using sex × genotype × treatment models with five Šídák-adjusted contrasts per endpoint. Brackets indicate within-genotype Model–Control or Nicotine–Model comparisons. Exact pooled-variance and Welch results are in Table P1. GSH is shown in Fig. 4D. *$P_{adj} < 0.05$; **$P_{adj} < 0.01$; ***$P_{adj} < 0.001$; ****$P_{adj} < 0.0001$; ns, $P_{adj} \geq 0.05$.

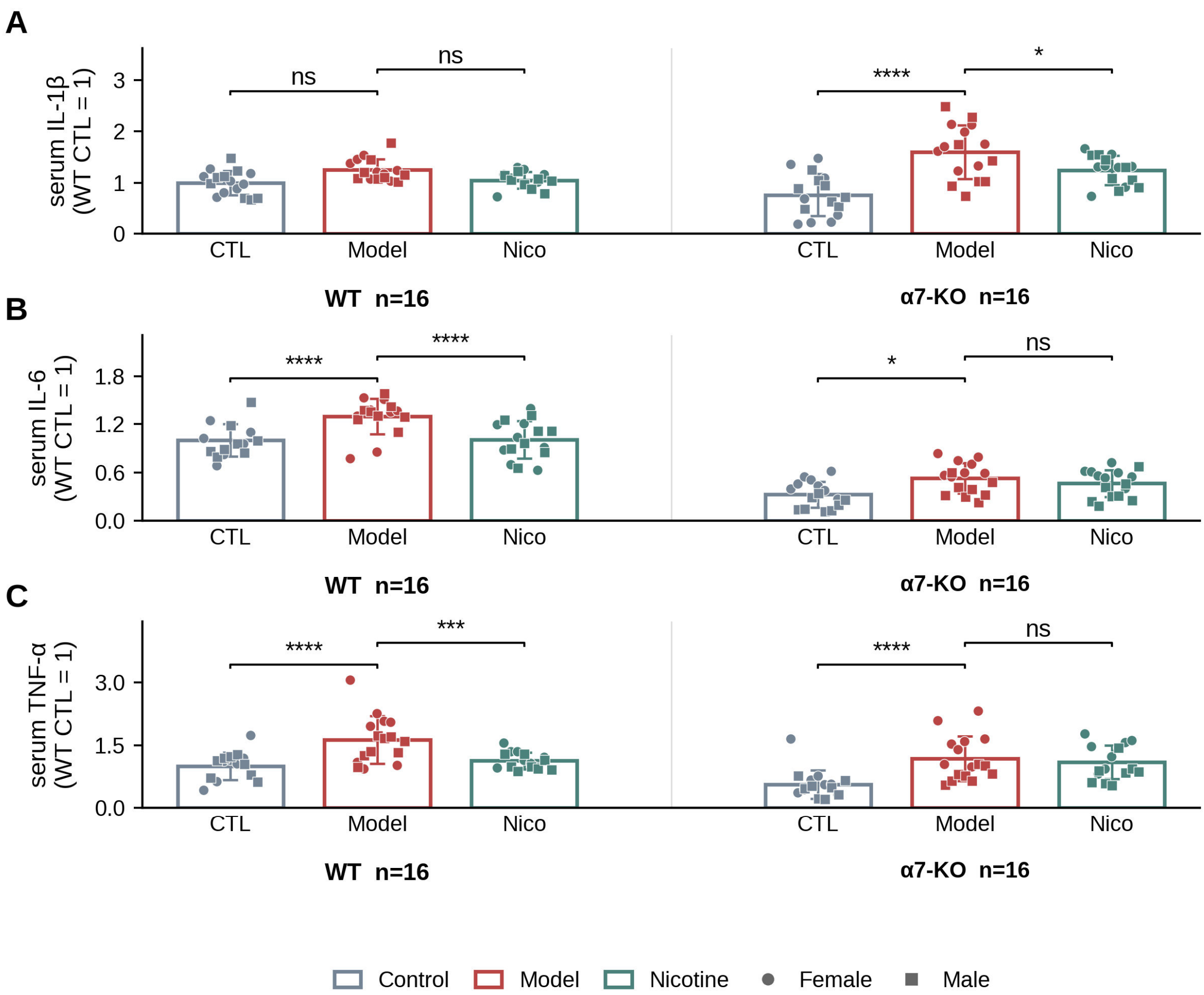

Supplementary Figure S3. Serum IL-1β, IL-6, and TNF-α. Display values were normalized to the same-sex WT Control mean; n = 16 rats per genotype and treatment. Bars show mean ± SD; circles denote females and squares denote males. Unscaled measurements were analyzed using sex × genotype × treatment models with five Šídák-adjusted contrasts per endpoint. Brackets indicate within-genotype Model–Control or Nicotine–Model comparisons. Exact pooled-variance and Welch results are in Table P1. $^*P_{adj} < 0.05$; $^{**}P_{adj} < 0.01$; $^{***}P_{adj} < 0.001$; $^{****}P_{adj} < 0.0001$; ns, $P_{adj} \geq 0.05$.

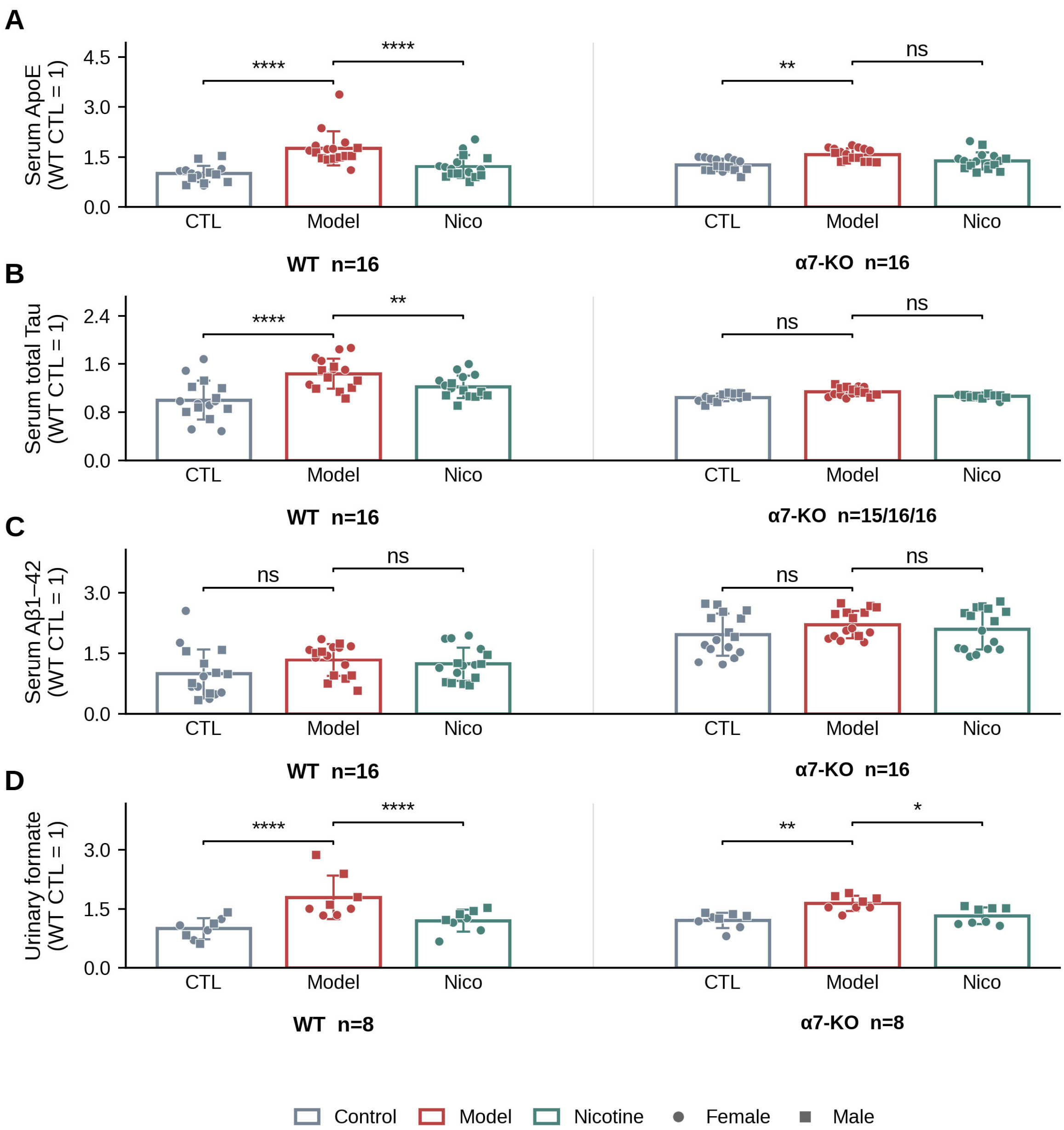

Supplementary Figure S4. Serum ApoE, total Tau, and Aβ1–42, and urinary formate. Display values were normalized to the same-sex WT Control mean; analyses used unscaled measurements. Bars show mean ± SD; circles denote females and squares denote males. Serum groups contained n = 16 rats per genotype and treatment, except KO Control Tau, n = 15. Urinary formate groups contained n = 8. Sex × genotype × treatment models were followed by five Šídák-adjusted contrasts per endpoint. Formate ratios are dimensionless; urine measurements were not normalized to creatinine or specific gravity. Exact results are in Table P1. $*P_{adj} < 0.05$; $**P_{adj} < 0.01$; $***P_{adj} < 0.001$; $****P_{adj} < 0.0001$; ns, $P_{adj} \geq 0.05$.

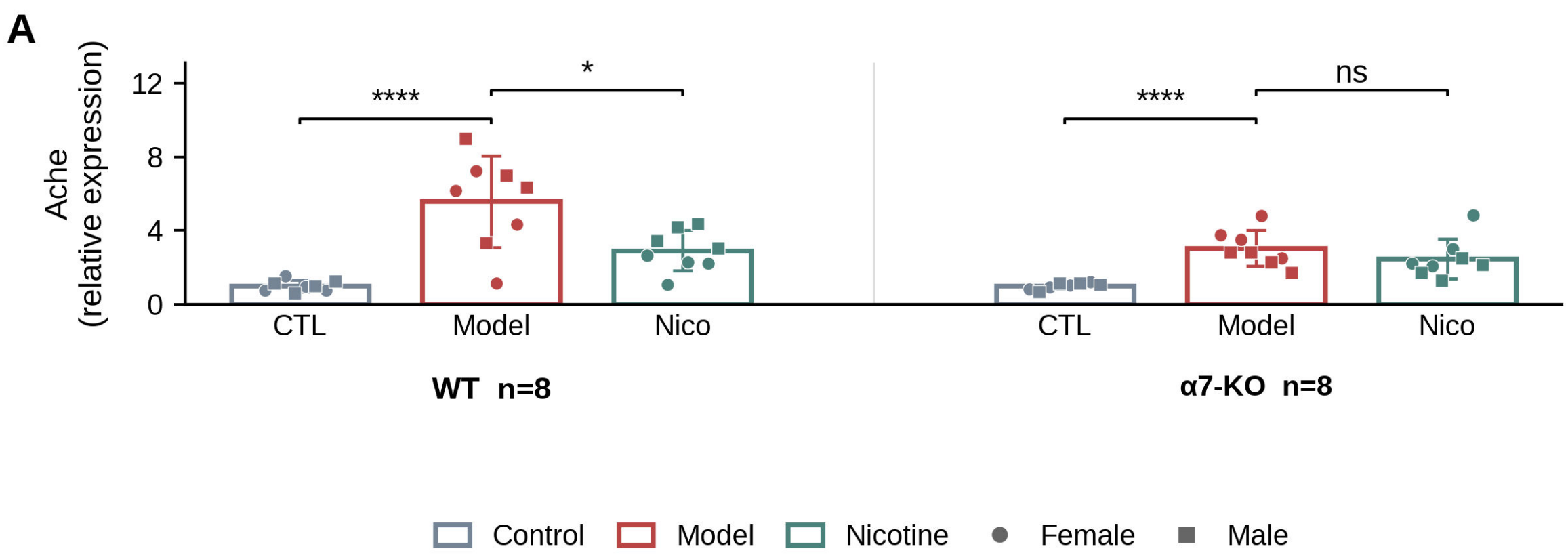

Supplementary Figure S5. Hippocampal *Ache* expression. Relative mRNA expression is shown for n = 8 rats per genotype and treatment, comprising four females and four males. Bars show mean ± SD; circles denote females and squares denote males. Values were calibrated to the common Control within each gene, sex, and genotype. A sex-adjusted model on $\log_2$(relative expression) was followed by four sex-averaged contrasts with Šídák adjustment. Exact results are in Table P4. The analysis concerns within-genotype treatment responses. $*P_{adj} < 0.05$; $**P_{adj} < 0.01$; $***P_{adj} < 0.001$; $****P_{adj} < 0.0001$; ns, $P_{adj} \geq 0.05$.

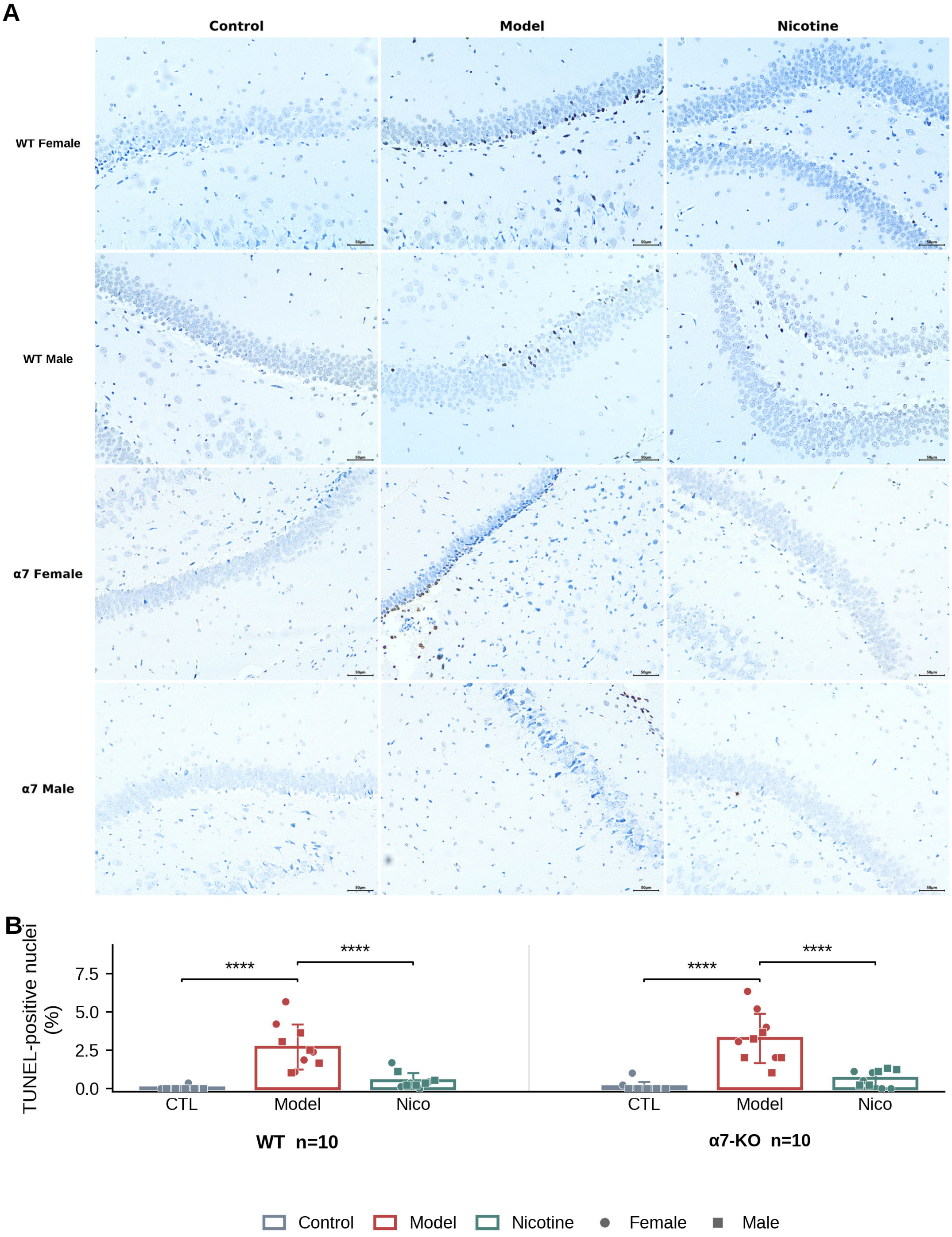

Supplementary Figure S6. Hippocampal TUNEL-positive nuclei. (A) Representative staining. Columns show Control, Model, and Nicotine; rows show female WT, male WT, female α7-KO, and male α7-KO rats. Scale bars, 50 µm. (B) Percentage of TUNEL-positive nuclei; n = 10 rats per genotype and treatment. Bars show mean ± SD; circles denote females and squares denote males. Percentages were analyzed using a sex × genotype × treatment model with five Šídák-adjusted contrasts. Brackets indicate within-genotype comparisons. Exact pooled-variance and Welch results are in Table P1. TUNEL labeling was not assigned to a specific cell type. *$P_{adj} < 0.05$; **$P_{adj} < 0.01$; ***$P_{adj} < 0.001$; ****$P_{adj} < 0.0001$; ns, $P_{adj} \geq 0.05$.

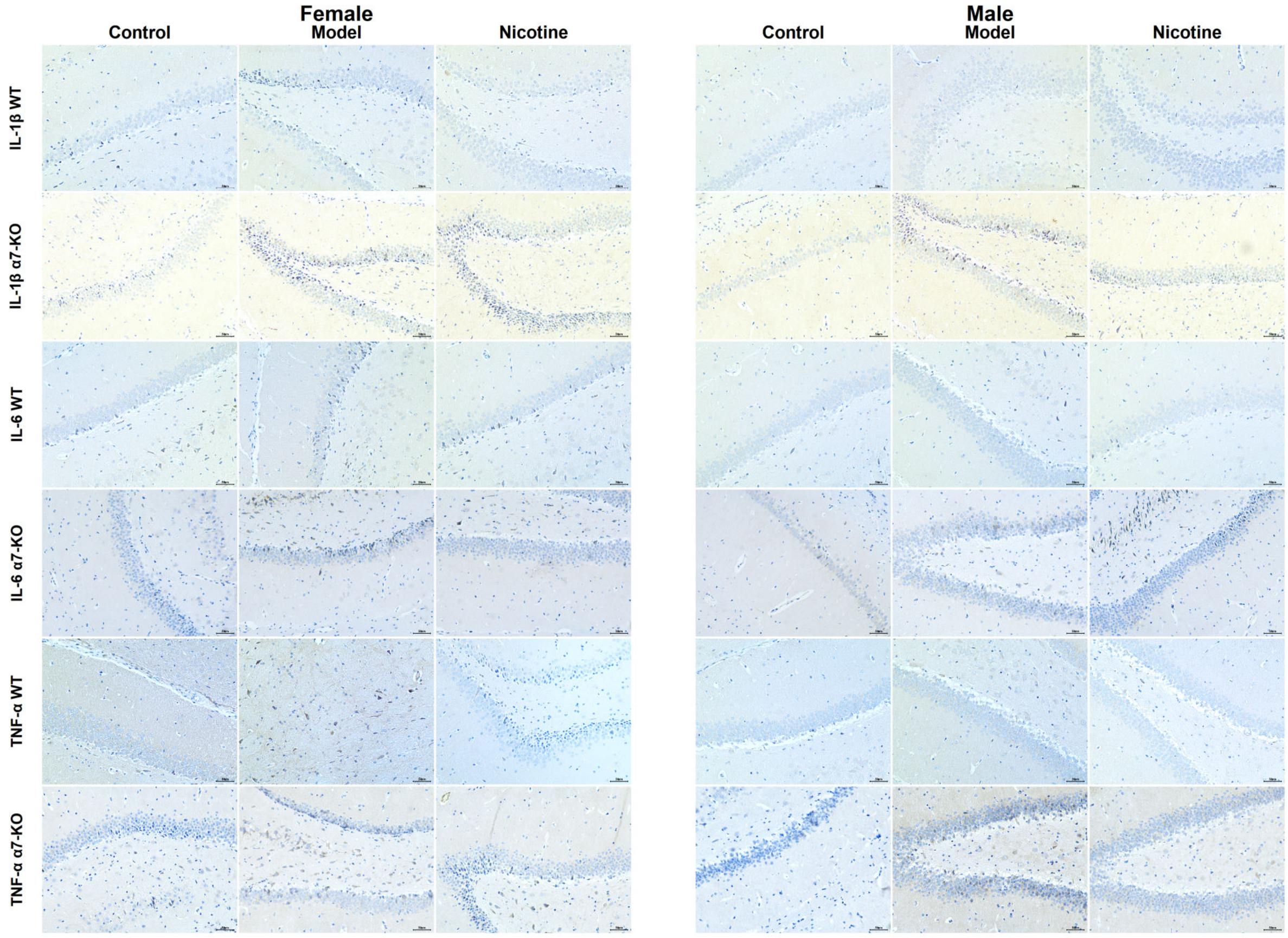

Supplementary Figure S7. Hippocampal cytokine immunohistochemistry. The left three columns show female rats and the right three show male rats, with Control, Model, and Nicotine within each sex. Successive pairs of rows show WT and α7-KO rats for IL-1β, IL-6, and TNF-α. Figure 3A contains the corresponding female WT fields. Scale bars, 50 μm.

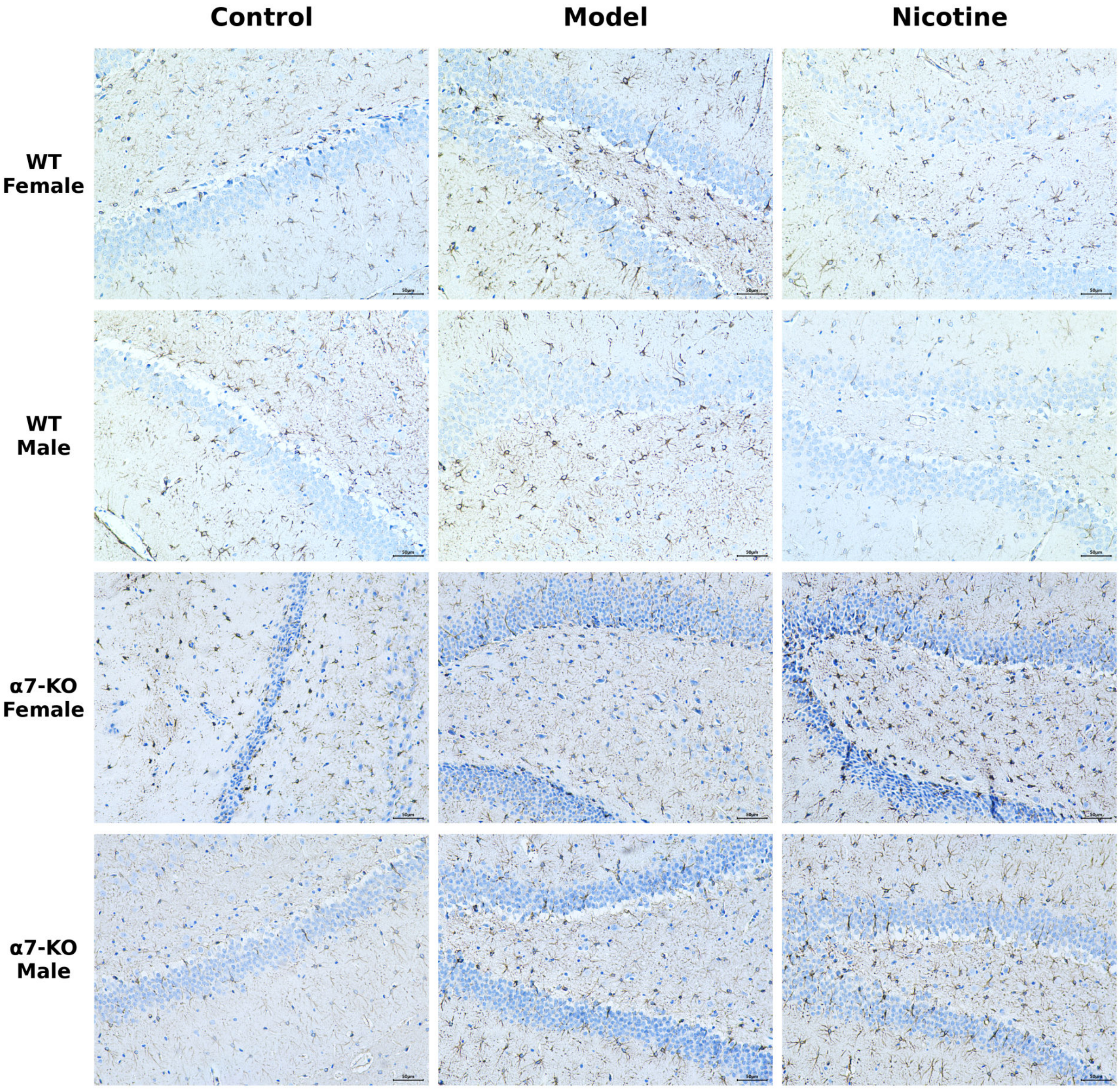

Supplementary Figure S8. Hippocampal GFAP immunohistochemistry. Columns show Control, Model, and Nicotine. Rows show female WT, male WT, female α7-KO, and male α7-KO rats. Corresponding female WT fields are included in Fig. 4A. Scale bars, 50 μm.

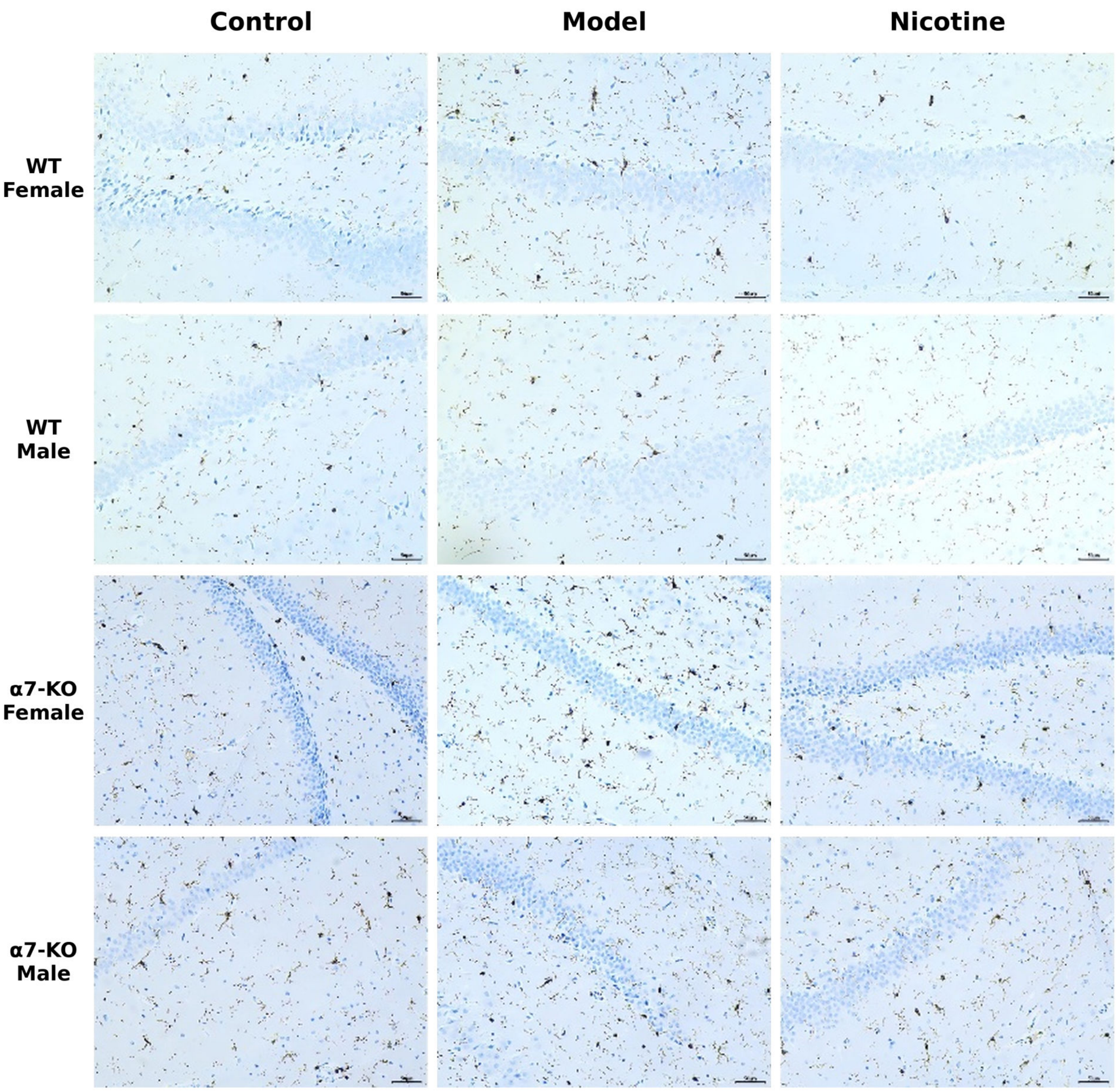

Supplementary Figure S9. Hippocampal IBA1 immunohistochemistry. Columns show Control, Model, and Nicotine. Rows show female WT, male WT, female α7-KO, and male α7-KO rats. Corresponding female WT fields are included in Fig. 4A. Scale bars, 50 μm.

Supplementary Table S1. Reagents and study applications

Manufacturers, identifiers, and study applications are listed below. All six IHC primary antibodies were used at 1:100.

Product information and supporting documentation are provided in Supplementary Data 1.

**Reagent**	**Supplier / identifier / formulation**	**Study application**
GFAP	Proteintech 16825-1-AP; RRID:AB_2109646	Hippocampal IHC; 1:100.
IBA1	Proteintech 10904-1-AP; RRID:AB_2224377	Hippocampal IHC; 1:100.
IL-1β	Abcam ab283818; RRID:AB_3076536	Hippocampal IHC; 1:100.
IL-6	Abcam ab9324; RRID:AB_307175	Hippocampal IHC; 1:100.
Aβ IHC	Abcam ab201060; RRID:AB_2818982	Tissue IHC; 1:100. Distinct from the serum Aβ1–42 ELISA.
TNF-α IHC	Abcam ab307164; RRID:AB_2941996	Hippocampal IHC; 1:100.
SA-β-gal	Beyotime C0602	Tissue and culture SA-β-gal staining.
D-galactose, culture	Sigma-Aldrich V900922-100G	Culture concentration-response series, 0–250 mM; co-exposure condition, 200 mM.
D-galactose, animal	Sigma-Aldrich; lot BCBZ9102	180 mg/kg/day, intraperitoneally for 49 days.
Urinary formate	Abcam ab111748	Urinary formate assay; displayed as same-sex WT Control ratios. No creatinine or specific-gravity normalization.
Serum SOD / GSH / MDA	Shanghai Enzyme-linked Biotechnology: Ml059387 / ML531010 / ml640319	Serum measurements: SOD, ng/mL; GSH, mmol/L; MDA, nmol/mL.
Serum IL-1β / IL-6 / TNF-α	Shanghai Enzyme-linked Biotechnology: Ml003057 / Ml064292 / Ml002859	Serum ELISA; distinct from tissue-IHC antibodies.
Serum ApoE / total Tau	Shanghai Enzyme-linked Biotechnology: ml003325 / ml038138	Serum ApoE and total Tau ELISA.
Serum Aβ1–42	Shanghai Enzyme-linked Biotechnology: ml003356	Serum Aβ1–42 ELISA.
RNA extraction for qPCR	Takara Bio 9767	TaKaRa MiniBEST Universal RNA Extraction Kit.
Reverse transcription	Takara Bio RR047A	PrimeScript RT reagent Kit with gDNA Eraser.
qPCR master mix	Takara Bio RR820A	TB Green Premix Ex Taq II (Tli RNaseH Plus).
Nicotine	Free-base formulation; stock, 1 mg/mL in normal saline. Same reagent used in both experimental systems.	Animal: 0.5 mg/kg/day, intraperitoneally for 49 days. Culture: 50–200 μM during 200 mM D-galactose exposure.
TUNEL detection kit	Sangon Biotech	Hippocampal in situ TUNEL staining; DAB visualization and hematoxylin counterstaining.

Supplementary Table S2. qPCR primer sequences

Sequences are shown in the 5′–3′ direction. F and R denote forward and reverse primers, respectively. The six primer pairs are listed below. Tm values refer to primer melting temperatures, not amplification efficiencies. Amplification and melting curves are shown in Fig. S17.

**Gene**	**Forward, 5′–3′**	**Reverse, 5′–3′**	**Accession; primer F/R Tm (°C)**
App	CCTTACCGGTGCCTAGTTGG	GAATCGATGCTGTCGCTCTC	NM_019288.2<br>Tm: 58.4 / 56.1
Psen1	CGGAAGGATGGGCAGCTAAT	CACGGCGACATTGTAGGTCT	NM_019163.4<br>Tm: 57.7 / 57.6
Bace1	TTCATGTTGCCACTCTGCCT	CCTTGCAAGCCCTGTAATCCA	NM_019204.2<br>Tm: 57.0 / 57.9
Ache	TGAGAGATGCCATGAGTGCG	GGGGAGCCCAAAGATGAACT	BC094521.1<br>Tm: 57.7 / 57.6
Chrna7 (α7-nAChR)	TGTCAGGTACAACGGCACTG	CCATCGCGCTCATGATTTCC	Tm: 57.8 / 57.1
Gapdh	TCTCTGCTCCTCCCTGTTCT	CCGATACGGCCAAATCCGTT	NM_017008.4<br>Tm: 56.1 / 55.5

Supplementary Table S3. RNA-seq sample identifiers

The 36 identifiers correspond to six independent WT rats in each sex × treatment group. Sequencing used DNBSEQ-T7 with paired-end 150-bp reads. Sequence data are deposited under BioProject PRJCA074229 and GSA CRA050070.

**Sample identifier**	**Sample label**
WHT2510027979	control-1-F
WHT2510027980	control-2-F
WHT2510027981	control-3-F
WHT2510027982	control-4-F
WHT2510027983	control-5-F
WHT2510027984	control-6-F
WHT2510027985	Model-1-F
WHT2510027986	Model-2-F
WHT2510027987	Model-3-F
WHT2510027988	Model-4-F
WHT2510027989	Model-5-F
WHT2510027990	Model-6-F
WHT2510027991	Nicotine-1-F
WHT2510027992	Nicotine-2-F
WHT2510027993	Nicotine-3-F
WHT2510027994	Nicotine-4-F
WHT2510027995	Nicotine-5-F
WHT2510027996	Nicotine-6-F
WHT2510027997	control-1-M
WHT2510027998	control-2-M
WHT2510027999	control-3-M
WHT2510028000	control-4-M
WHT2510028001	control-5-M
WHT2510028002	control-6-M
WHT2510028003	Model-1-M
WHT2510028004	Model-2-M
WHT2510028005	Model-3-M
WHT2510028006	Model-4-M
WHT2510028007	Model-5-M
WHT2510028008	Model-6-M
WHT2510028009	Nicotine-1-M
WHT2510028010	Nicotine-2-M
WHT2510028011	Nicotine-3-M
WHT2510028012	Nicotine-4-M
WHT2510028013	Nicotine-5-M
WHT2510028014	Nicotine-6-M

Statistical tables and source data

Table P1: Table_P1_pooled_sex_adjusted_contrasts.csv. Ninety-five fixed sex-averaged contrasts for 19 animal endpoints, with pooled-variance and Welch–Satterthwaite estimates, standard errors, degrees of freedom, nominal and Šídák-adjusted P values, confidence intervals and significance codes.

Table P2: Table_P2_sex_heterogeneity_tests.csv. Sex × treatment and sex × genotype × treatment heterogeneity tests for every endpoint, with Šídák adjustment across the two tests within each endpoint. These tests do not imply that female-versus-male pairwise comparisons are study objectives.

Table P3: Table_P3_pooled_group_summaries.csv. Sex-specific raw and display-scale group summaries underlying the pooled plots, including exact n, means and SDs.

Table P4: Table_P4_qPCR_pooled_sex_adjusted_contrasts.csv. Sex-averaged qPCR treatment contrasts on $\log_2$(relative expression), with four-comparison Šídák adjustment within each gene. The source values retain separate WT and KO Control calibration.

Table P5: Table_P5_tissue_SA_pooled_sex_adjusted_contrasts.csv. Sex-averaged tissue SA-β-gal Model–Control and Nicotine–Model contrasts, with two-comparison Šídák adjustment.

Table P6: Table_P6_joint_factorial_omnibus.csv. Partial F tests for all seven terms of the joint sex × genotype × treatment model, with degrees of freedom, nominal P values and partial η^2. These omnibus tests do not replace the five fixed treatment contrasts.

Table P7: Table_P7_combined_display_group_summaries.csv. The exact combined-sex sample sizes, numbers of females and males, and arithmetic means and SDs on raw and displayed scales.

Animal models use unscaled measurements, whereas the non-qPCR plots display ratios relative to the same-sex WT Control mean. TUNEL results remain percentages. qPCR uses separate gene-, sex-, and genotype-specific Control calibrators and is analyzed on the $\log_2$ relative-expression scale. Culture concentration-response analyses and representative-field counts are separate analyses.

Sex-stratified sensitivity analyses (Tables S4–S8)

Tables S4–S8 provide sex-stratified sensitivity analyses. Table S4 reports genotype, treatment, and interaction F tests within each sex, with adjustment across three tests. Tables S5 and S6 use a five-comparison Šídák family within each endpoint and sex; Table S6 reports the direct WT–KO difference in the Nicotine–Model response. Table S7 provides group n, mean, SD, and SEM; Table S8 provides nominal diagnostic tests. Main-figure symbols correspond to the sex-averaged analyses rather than these sensitivity analyses.

Direct genotype-response sensitivity summary (Table S6)

Estimates and confidence intervals retain the endpoint-specific raw units given in Table S7; these are sex-stratified sensitivity results, not main-figure comparisons.

**Endpoint / sex**	**WT–KO response difference**	**Simultaneous 95% CI**	**P_{adj}**	**Welch P_{adj}**
Latency to first target entry / Female	-15.07	-38.37 to 8.236	0.369	0.611
Latency to first target entry / Male	-5.025	-22.16 to 12.11	0.941	0.774
platform-site crossings / Female	2.75	-1.429 to 6.929	0.351	0.159
platform-site crossings / Male	3.292	-0.08259 to 6.666	0.0587	0.0379
SOD / Female	0.167	-5.082 to 5.416	1	1
SOD / Male	1.375	-3.403 to 6.152	0.947	0.905
MDA / Female	-4.719	-14.44 to 5.005	0.67	0.749
MDA / Male	-2.485	-11.12 to 6.147	0.946	0.965
GSH / Female	36.11	-18.45 to 90.68	0.349	0.246

Endpoint / sex	WT–KO response difference	Simultaneous 95% CI	P_{adj}	Welch P_{adj}
GSH / Male	55.28	-14.54 to 125.1	0.181	0.184
serum IL-1β / Female	6.351	-7.152 to 19.85	0.698	0.536
serum IL-1β / Male	0.7423	-15.48 to 16.97	1	1
serum IL-6 / Female	-11.91	-37.85 to 14.03	0.718	0.796
serum IL-6 / Male	-27.24	-56.7 to 2.219	0.0818	0.0897
serum TNF-α / Female	-21.06	-68.65 to 26.53	0.747	0.792
serum TNF-α / Male	-32.58	-64.13 to -1.037	0.04	0.0526
hippocampal IL-1β IOD / Female	-8.51	-333.1 to 316.1	1	1
hippocampal IL-1β IOD / Male	-1.988	-360.4 to 356.4	1	1
hippocampal IL-6 IOD / Female	-136.4	-467.1 to 194.4	0.781	0.829
hippocampal IL-6 IOD / Male	63	-245.4 to 371.4	0.986	0.994
hippocampal TNF-α IOD / Female	-133.2	-401 to 134.6	0.625	0.763
hippocampal TNF-α IOD / Male	160.6	-168 to 489.2	0.642	0.819
GFAP IOD / Female	-779.7	-3755 to 2196	0.959	0.908
GFAP IOD / Male	-858.6	-3363 to 1646	0.883	0.887
IBA1 IOD / Female	-1113	-2031 to -196	0.0122	0.0194
IBA1 IOD / Male	-909.8	-2463 to 643.5	0.459	0.598
TUNEL-positive nuclei / Female	1.084	-1.675 to 3.843	0.812	0.912
TUNEL-positive nuclei / Male	-0.314	-1.974 to 1.346	0.99	0.996
Urinary formate / Female	-0.03361	-0.3637 to 0.2965	0.999	0.999
Urinary formate / Male	-0.2922	-0.7599 to 0.1756	0.376	0.615
ApoE / Female	-26.5	-71.62 to 18.61	0.477	0.68
ApoE / Male	-25.91	-60.37 to 8.56	0.225	0.213
Serum Aβ1–42 / Female	5.011	-11.88 to 21.9	0.939	0.733
Serum Aβ1–42 / Male	-7.199	-29.51 to 15.11	0.916	0.883
Tau / Female	-66.18	-207.6 to 75.28	0.702	0.356
Tau / Male	-45.25	-158 to 67.53	0.815	0.7
mean distance to target / Female	-11.04	-26.18 to 4.09	0.249	0.329
mean distance to target / Male	-3.989	-13.35 to 5.368	0.771	0.602

Supplementary Table S9. Sample-level RNA-seq quality control

Sample-level quality metrics for all 36 samples are provided in Table_S9_RNAseq_quality_metrics.csv, including clean yield, Q20, Q30, clean-read ratios, and genome-mapping rates. Filtering and mapping totals use their respective counting conventions. Sample identifiers are provided separately in Table S3. Complete expression, DEGseq, and enrichment results are supplied in Supplementary Data 1; figure-level gene lists and numerical summaries are supplied in Supplementary Data 3 and Table S14.

Supplementary Table S10. Descriptive SA-β-gal-positive cell-body counts

Each row represents one unique tissue or culture field. Clear blue or blue-green cell bodies were counted using software-assisted visual image annotation, excluding image labels and scale-bar regions. Weak staining, unresolved overlaps, and ambiguous deposits were classified separately as uncertain objects. These categories are mutually exclusive. Counts are descriptive field-level observations, distinct from animal- or donor-level positive-area measurements; no total-cell denominator or inferential P value is assigned. The shared Model field is counted once. Culture nicotine concentrations are in μM during exposure to 200 mM D-galactose; concentrations in the D-galactose series are in mM. The coordinate annotations are provided in Supplementary Data 2. This descriptive procedure was not independently validated against an expert-count reference.

Image	Material / condition	Clear positive	Uncertain
T01	tissue / Female Control	1	6
T02	tissue / Male Control	2	5
T03	tissue / Male Nicotine	7	9
T04	tissue / Female Model	27	14
T05	tissue / Female Nicotine	11	6
T06	tissue / Male Model	18	11
C01	culture / Nicotine 150	10	3
C02	culture / D-gal 200 (shared Model)	19	13
C03	culture / Nicotine-series Control	7	4
C04	culture / Nicotine 200	4	9
C05	culture / Nicotine 100	7	8
C06	culture / D-gal 150	11	8
C07	culture / D-gal 100	12	8
C08	culture / D-gal 250	9	4
C09	culture / Nicotine 50	14	6
C10	culture / D-gal 0	0	5
C11	culture / D-gal 50	9	7

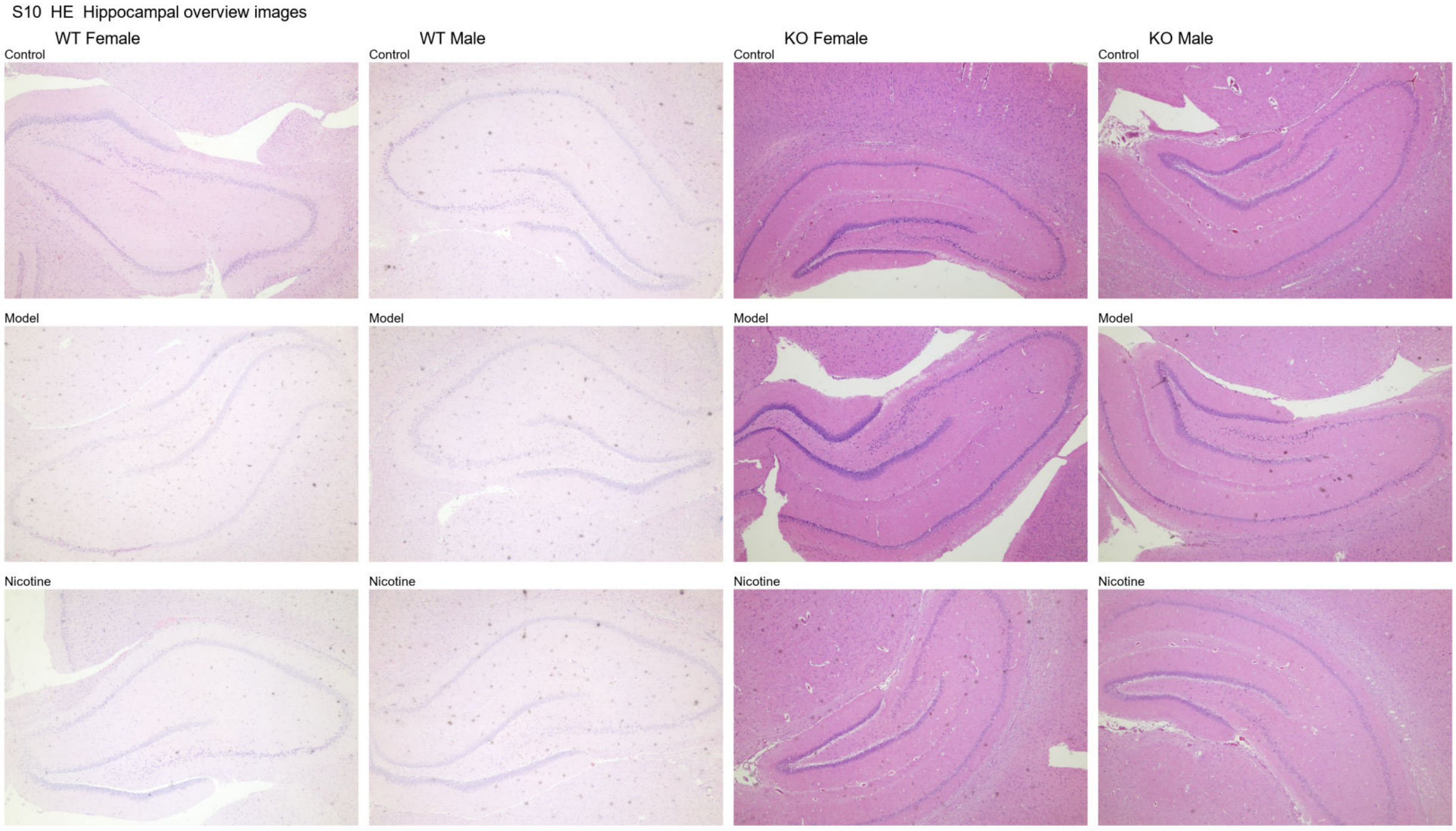

Supplementary Figure S10. Hippocampal overview images: H&E. Columns show female WT, male WT, female α7-KO, and male α7-KO rats; rows show Control, Model, and Nicotine. The overview images provide anatomical context and are not presented as registered locations of the high-magnification fields.

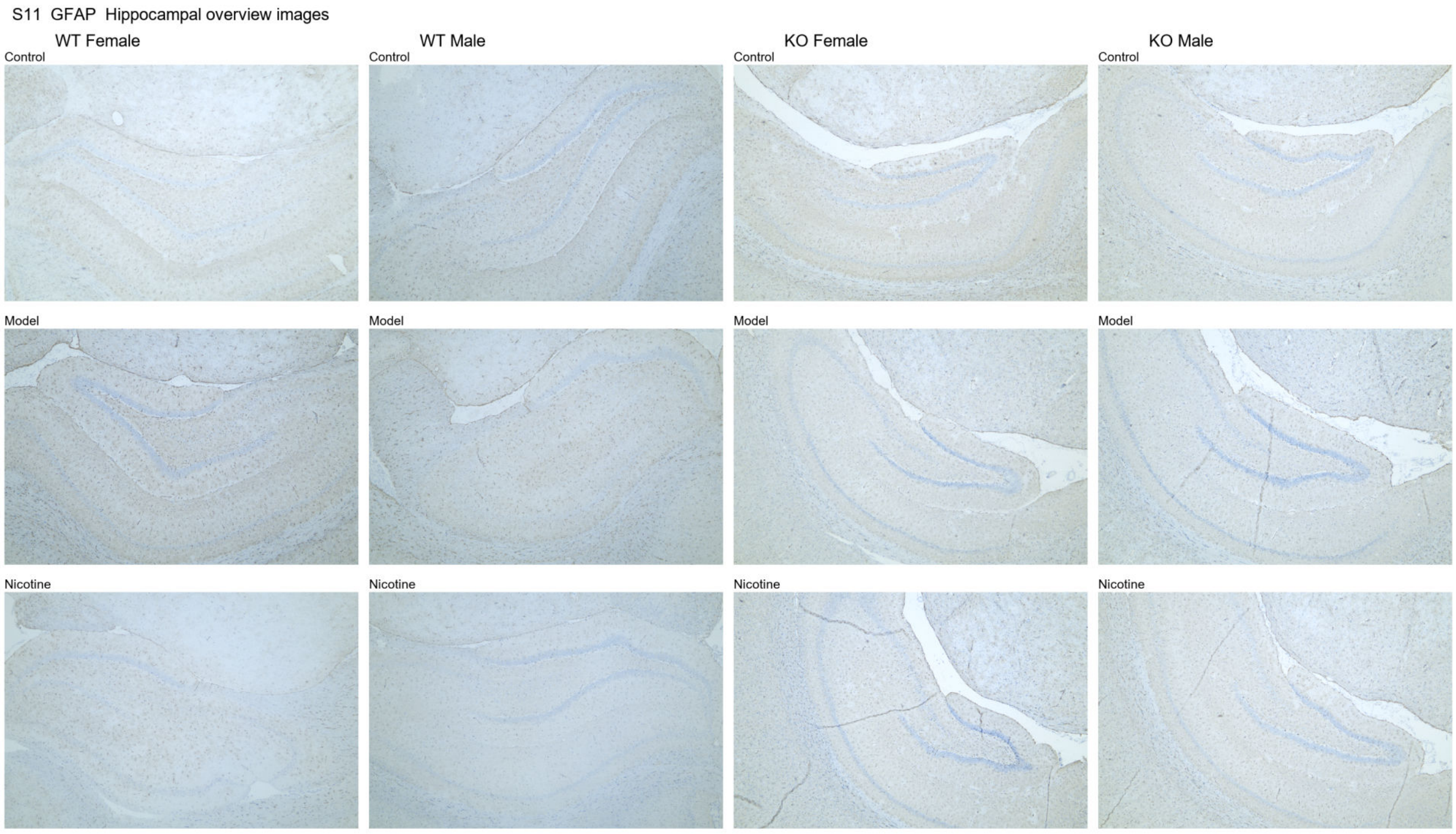

Supplementary Figure S11. Hippocampal overview images: GFAP. Columns show female WT, male WT, female α7-KO, and male α7-KO rats; rows show Control, Model, and Nicotine. The overview images provide anatomical context and are not presented as registered locations of the high-magnification fields.

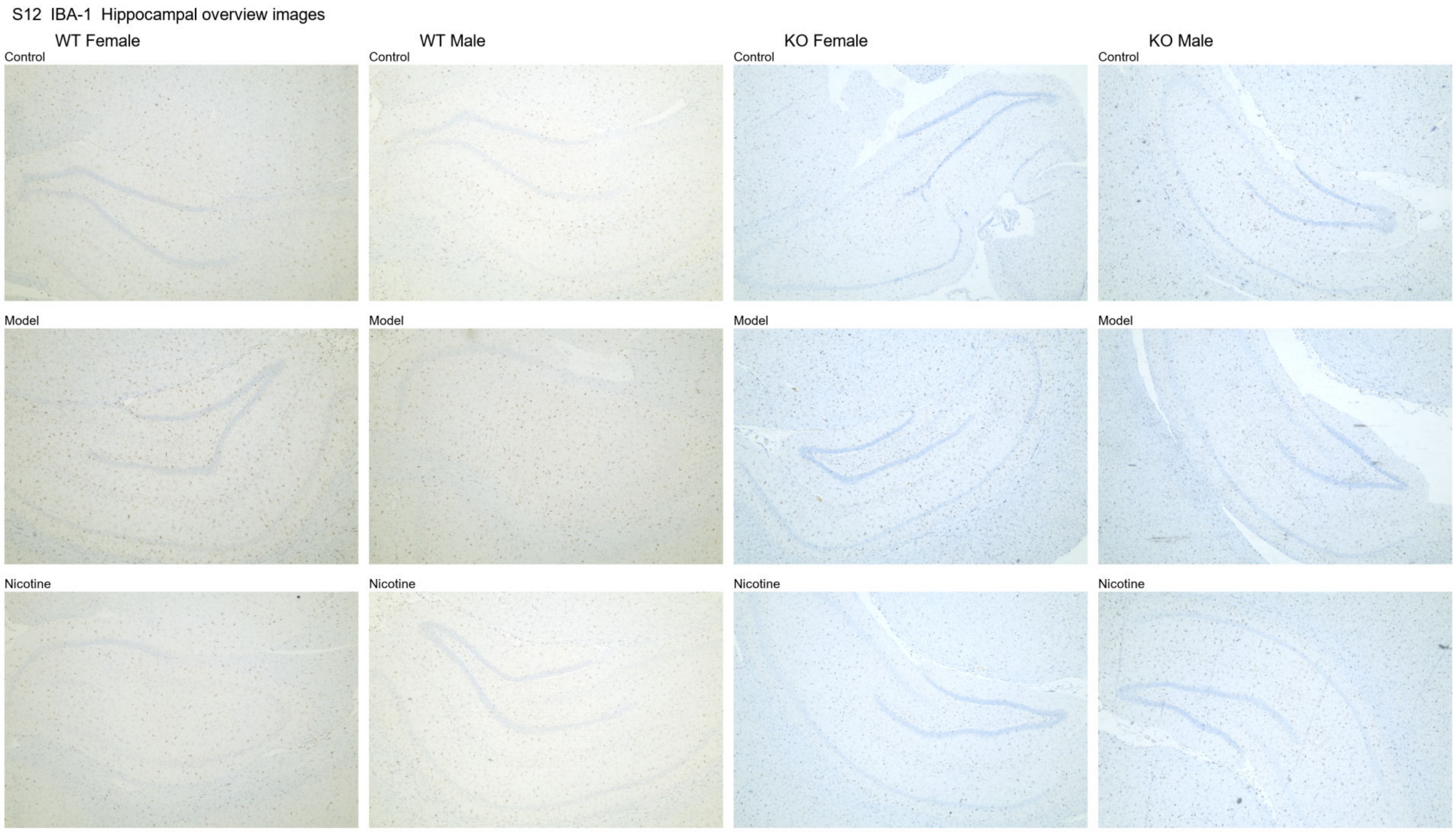

Supplementary Figure S12. Hippocampal overview images: IBA1. Columns show female WT, male WT, female α7-KO, and male α7-KO rats; rows show Control, Model, and Nicotine. The overview images provide anatomical context and are not presented as registered locations of the high-magnification fields.

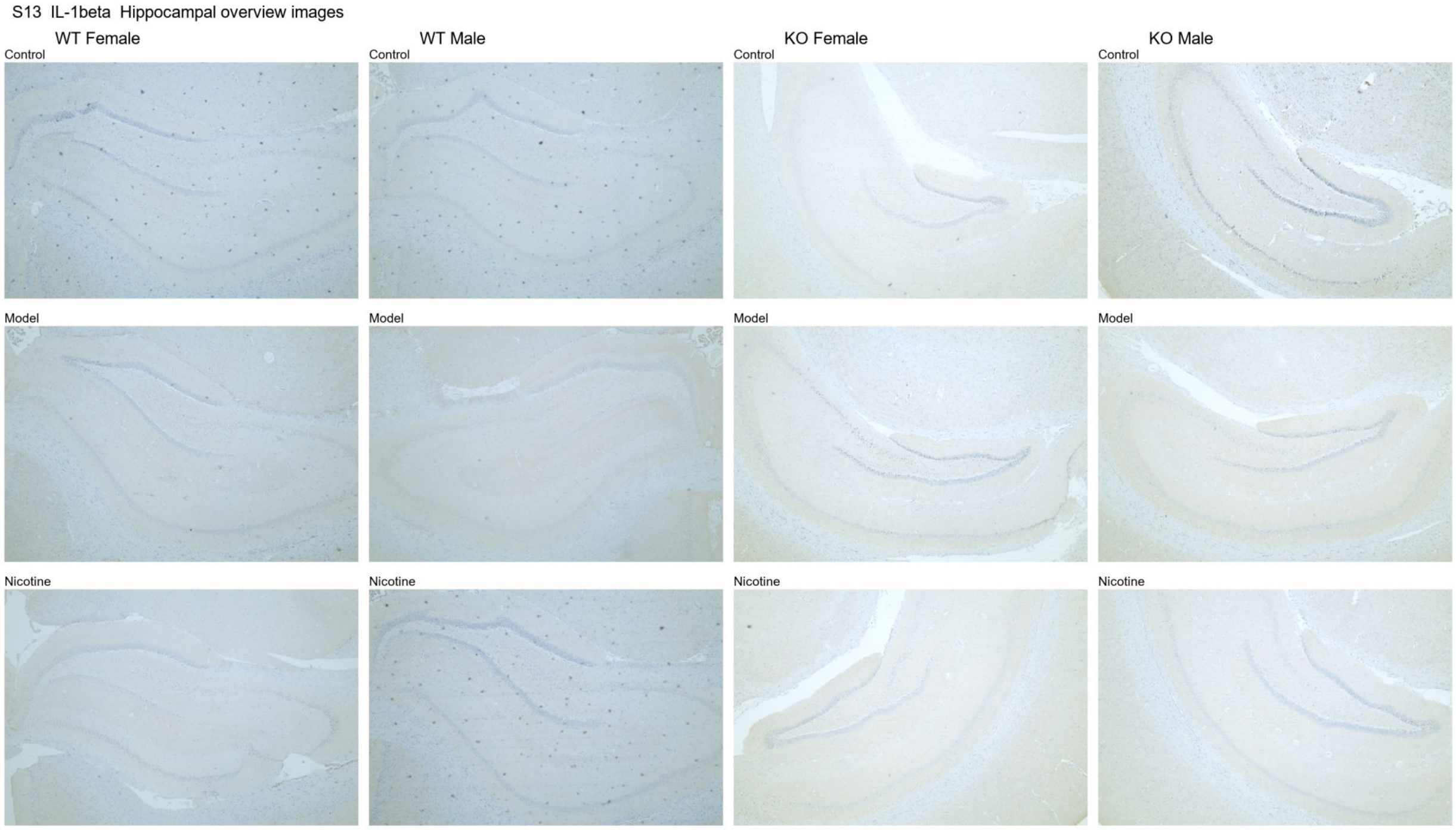

Supplementary Figure S13. Hippocampal overview images: IL-1β. Columns show female WT, male WT, female α7-KO, and male α7-KO rats; rows show Control, Model, and Nicotine. The overview images provide anatomical context and are not presented as registered locations of the high-magnification fields.

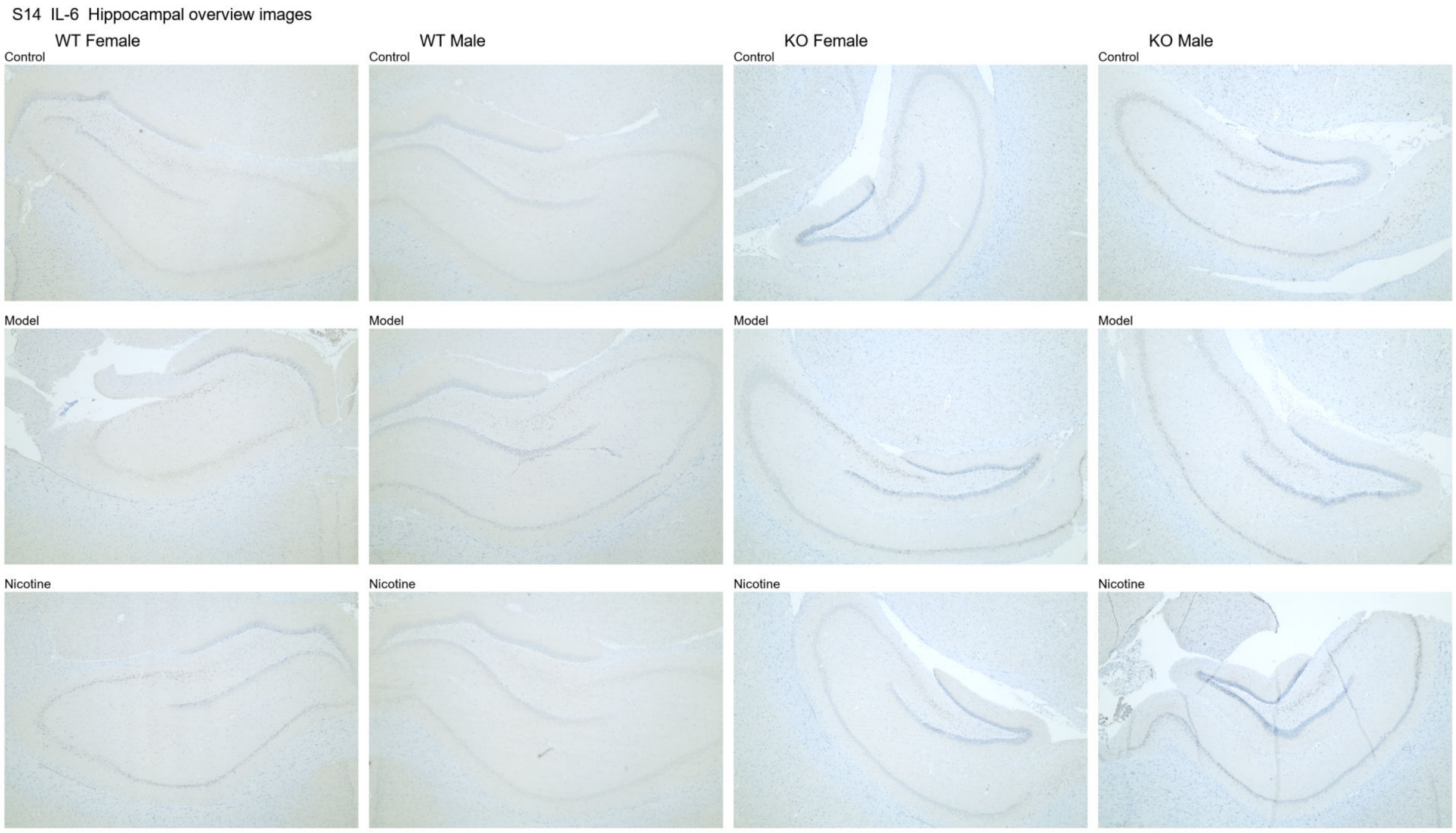

Supplementary Figure S14. Hippocampal overview images: IL-6. Columns show female WT, male WT, female α7-KO, and male α7-KO rats; rows show Control, Model, and Nicotine. The overview images provide anatomical context and are not presented as registered locations of the high-magnification fields.

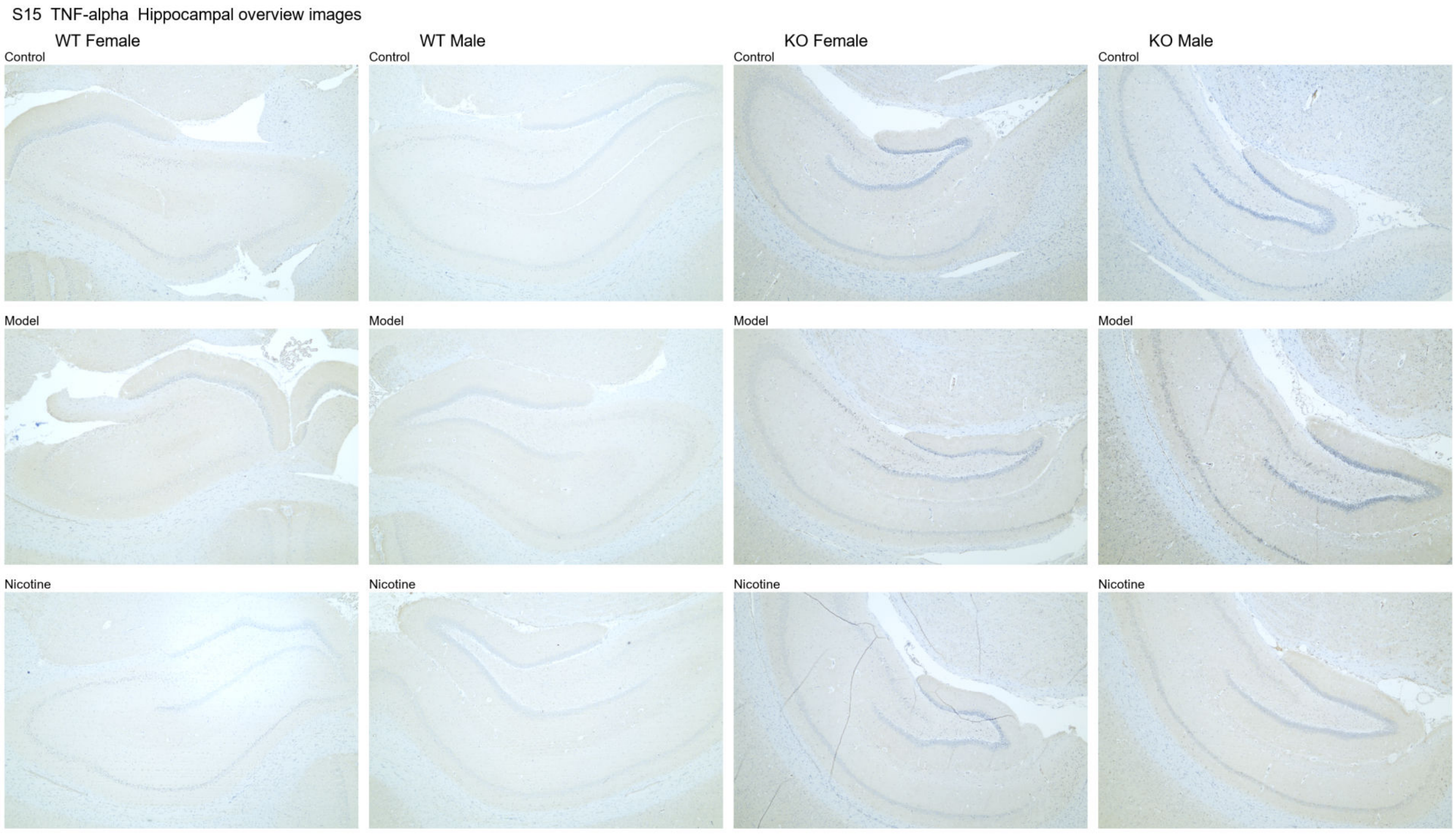

Supplementary Figure S15. Hippocampal overview images: TNF-α. Columns show female WT, male WT, female α7-KO, and male α7-KO rats; rows show Control, Model, and Nicotine. The overview images provide anatomical context and are not presented as registered locations of the high-magnification fields.

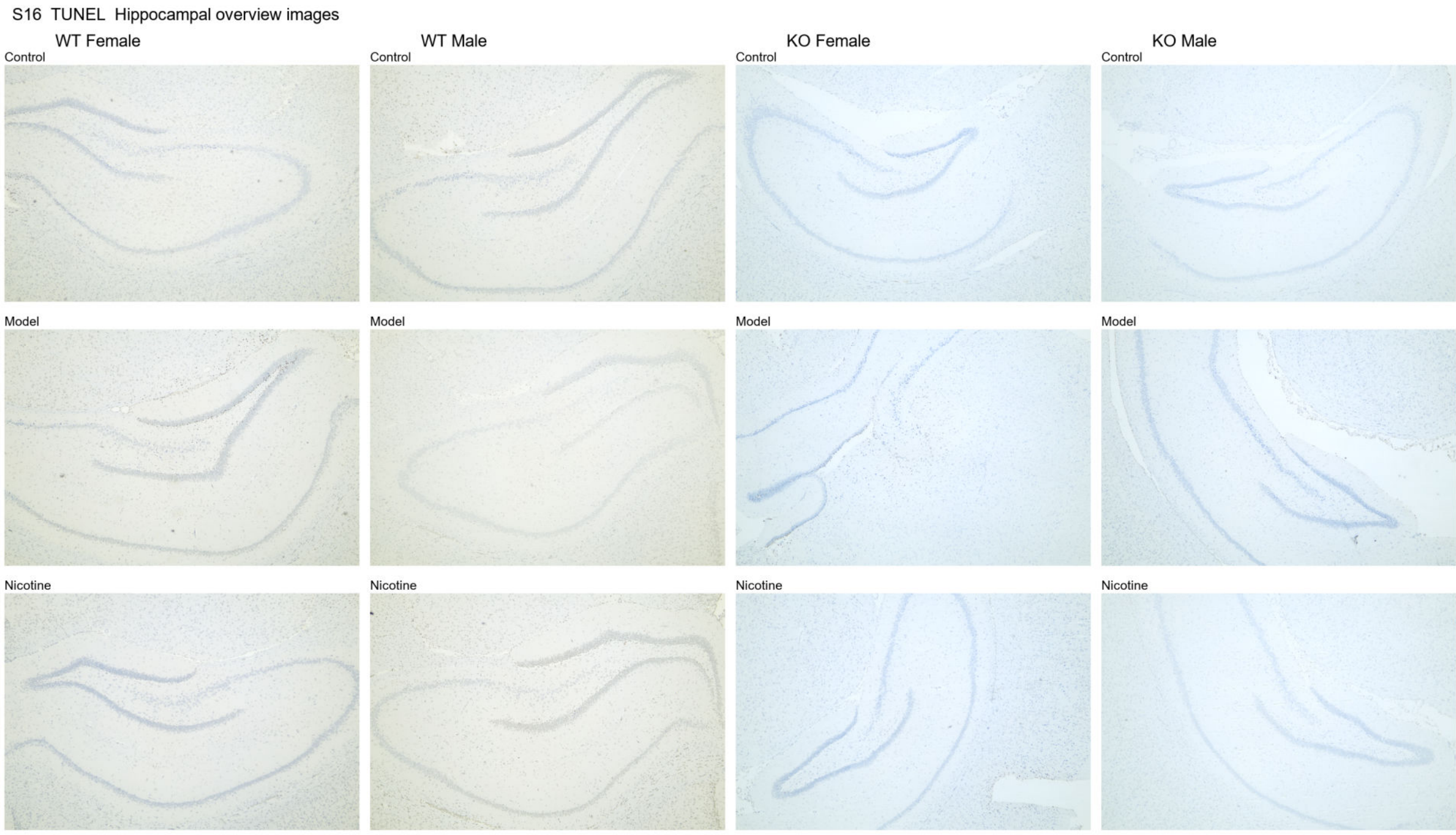

Supplementary Figure S16. Hippocampal overview images: TUNEL. Columns show female WT, male WT, female α7-KO, and male α7-KO rats; rows show Control, Model, and Nicotine. The overview images provide anatomical context and are not presented as registered locations of the high-magnification fields.

A Amplification curves

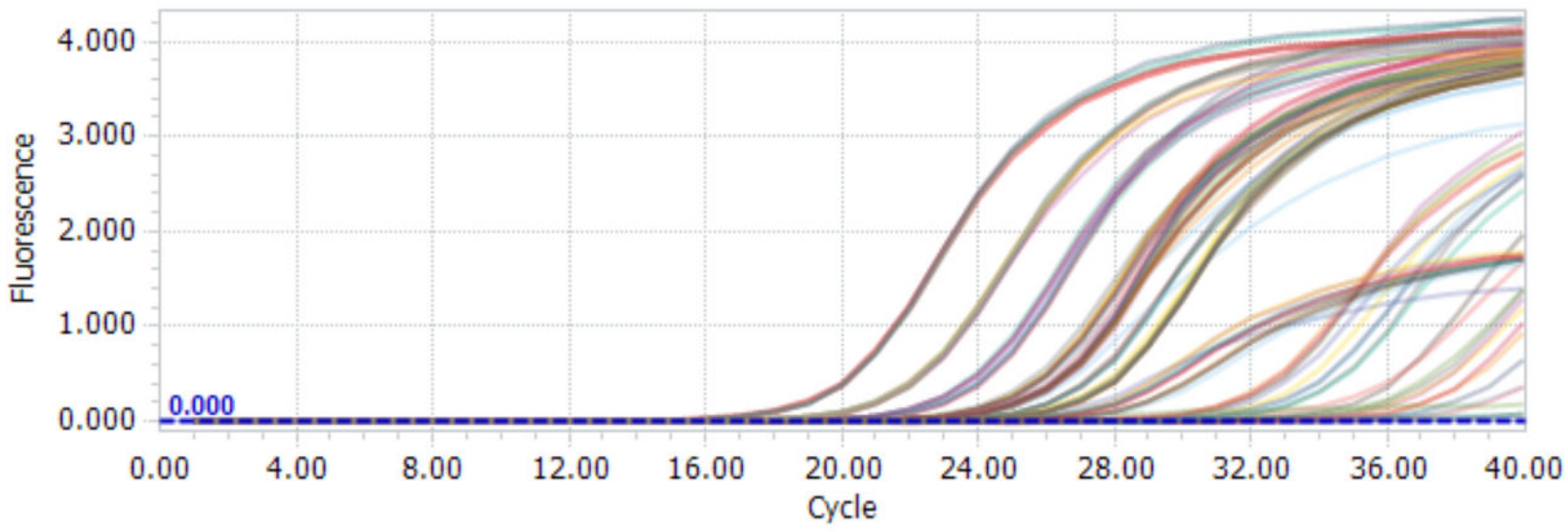

B Melting curves

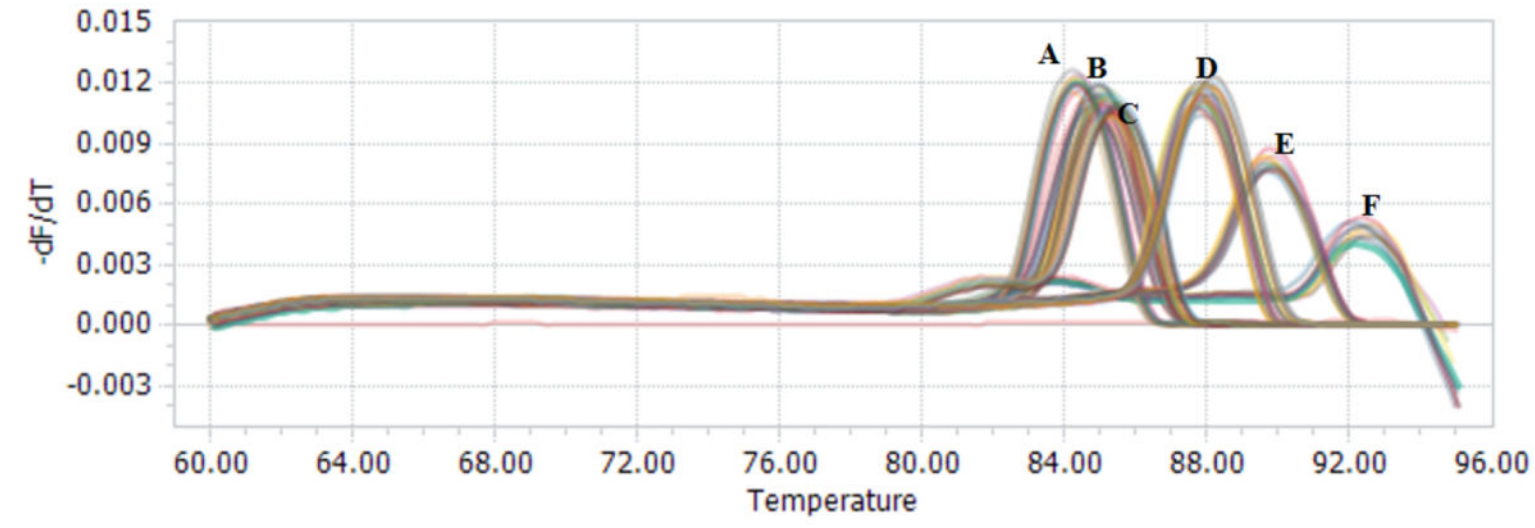

A：App B：Psen1 C：Bace1 D：GAPDH E:Achr F:Ache

Supplementary Figure S17. qPCR amplification and melting curves. (A) Amplification curves. (B) Melting curves with target labels: A, *App*; B, *Psen1*; C, *Bace1*; D, *Gapdh*; E, *Chrna7*; and F, *Ache*. The image label “Achr” corresponds to *Chrna7*, encoding α7-nAChR. These curves illustrate amplification and melting behavior; standard-curve amplification efficiencies are not derived from them.

Supplementary Table S11. Sex-stratified tissue SA-β-gal positive-area sensitivity comparisons

Six rats per treatment and sex. Within each sex, the Model–Control and Nicotine–Model contrasts were tested using Welch–Satterthwaite methods, with Šídák adjustment across two comparisons. These are sex-stratified sensitivity results. The joint sex × treatment analysis uses two sex-averaged contrasts with Šídák adjustment; its results appear in Table P5 and Fig. 1B.

**Sex**	**Comparison**	**Difference**	**Adjusted 95% CI**	**P_{adj}**	**Mark**
Female	Model-Control	0.2918	0.0729 to 0.5108	0.01688	*
Female	Nicotine-Model	-0.2618	-0.4801 to -0.0436	0.025351	*
Male	Model-Control	0.2738	0.1335 to 0.4141	0.0031678	**
Male	Nicotine-Model	-0.1960	-0.3556 to -0.0364	0.018114	*

Supplementary Table S12. Sex-stratified qPCR sensitivity comparisons

Four rats per treatment, sex and genotype. These sex-stratified sensitivity analyses use $\log_2$(relative expression). For each gene × sex × genotype stratum, pooled-variance contrasts use the residual variance across the three treatment groups (residual df = 9). Within each gene and sex, Šídák adjustment covers four contrasts: Model–Control and Nicotine–Model in WT and KO. The Welch sensitivity contrasts use the same four-comparison correction. The joint sex-adjusted qPCR analysis is reported separately in Table P4. WT and KO use separate Control calibrators; inference is restricted to within-genotype treatment contrasts. F, female; M, male; MC, Model–Control; NM, Nicotine–Model. *$P_{adj} < 0.05$; **$P_{adj} < 0.01$; ***$P_{adj} < 0.001$; ****$P_{adj} < 0.0001$; ns, $P_{adj} \geq 0.05$.

**Gene**	**Sex/type**	**Test**	**P_{adj}**	**Welch P_{adj}**	**Mark**
App	F/WT	MC	0.00060784	0.0033249	***
App	F/WT	NM	0.020321	0.097093	*
App	F/KO	MC	0.00092531	0.013277	***
App	F/KO	NM	0.95241	0.89825	ns
App	M/WT	MC	0.0030274	0.01069	**
App	M/WT	NM	0.36611	0.47854	ns
App	M/KO	MC	0.0094321	0.06339	**
App	M/KO	NM	0.78925	0.57871	ns
Bace1	F/WT	MC	0.00042926	0.0023713	***
Bace1	F/WT	NM	0.010551	0.065837	*
Bace1	F/KO	MC	0.0014507	0.01816	**
Bace1	F/KO	NM	0.8773	0.75989	ns
Bace1	M/WT	MC	8.1607e-05	0.0019003	****
Bace1	M/WT	NM	0.030476	0.08647	*
Bace1	M/KO	MC	0.03532	0.024532	*
Bace1	M/KO	NM	0.99384	0.99723	ns
Psen1	F/WT	MC	0.0008828	0.0020993	***
Psen1	F/WT	NM	0.0063941	0.070836	**
Psen1	F/KO	MC	0.006002	0.033867	**
Psen1	F/KO	NM	0.99945	0.99942	ns
Psen1	M/WT	MC	0.00050735	0.01094	***
Psen1	M/WT	NM	0.35256	0.51536	ns
Psen1	M/KO	MC	0.0054814	0.013274	**
Psen1	M/KO	NM	0.96345	0.97013	ns
Chrna7	F/WT	MC	0.34775	0.51136	ns
Chrna7	F/WT	NM	0.0044698	0.016851	**
Chrna7	F/KO	MC	0.97693	0.98694	ns
Chrna7	F/KO	NM	0.98777	0.98814	ns
Chrna7	M/WT	MC	0.99512	0.99757	ns
Chrna7	M/WT	NM	0.16135	0.30214	ns
Chrna7	M/KO	MC	0.62935	0.7407	ns
Chrna7	M/KO	NM	0.80007	0.79707	ns
Ache	F/WT	MC	0.027533	0.13503	*
Ache	F/WT	NM	0.40354	0.59949	ns
Ache	F/KO	MC	0.00059272	0.0021851	***
Ache	F/KO	NM	0.78441	0.87193	ns
Ache	M/WT	MC	7.939e-05	0.0024243	****
Ache	M/WT	NM	0.2163	0.34158	ns
Ache	M/KO	MC	0.0038538	0.0094754	**
Ache	M/KO	NM	0.62078	0.6543	ns

Supplementary Table S13. Culture SA-β-gal concentration-response comparisons

Four independent donor-derived wells were included per condition, with one well and one plotted data point per donor mouse. Each six-condition series was analyzed using a separate one-way ANOVA. Two-sided Dunnett tests compared five nonzero D-galactose concentrations with 0 mM, or four nicotine concentrations with the Model condition. Residual mean-square errors and 18 residual degrees of freedom were obtained from all six conditions, including the untreated control in the nicotine series. Exact statistics and simultaneous intervals are provided in Supplementary Data 2.

Series	Comparison	n per condition	Dunnett P_{adj}	Symbol
D-galactose concentration	50 mM vs 0 mM	4	0.0187	*
D-galactose concentration	100 mM vs 0 mM	4	8.32×10^{-6}	****
D-galactose concentration	150 mM vs 0 mM	4	5.34×10^{-6}	****
D-galactose concentration	200 mM vs 0 mM	4	8.06×10^{-10}	****
D-galactose concentration	250 mM vs 0 mM	4	2.77×10^{-7}	****
Nicotine with 200 mM D-galactose	50 µM vs Model	4	1.81×10^{-7}	****
Nicotine with 200 mM D-galactose	100 µM vs Model	4	1.8×10^{-10}	****
Nicotine with 200 mM D-galactose	150 µM vs Model	4	9.49×10^{-15}	****
Nicotine with 200 mM D-galactose	200 µM vs Model	4	5.26×10^{-15}	****

Supplementary Table S14. Within-sex RNA-seq DEG counts

DEGs were defined using DEGseq $q < 0.05$ and $|\log_2$ fold change$| \geq 1$. Each comparison includes six independent WT rats per treatment within the indicated sex. Increased and decreased expression refer to Model relative to Control or Nicotine relative to Model. These are within-sex comparisons, not formal tests of a sex difference.

Sex	Comparison	Increased	Decreased	Total
Female	Model vs Control	702	172	874
Female	Nicotine vs Model	300	384	684
Male	Model vs Control	67	146	213
Male	Nicotine vs Model	128	272	400

The female intersection contains 288 genes and the union contains 1,270. Among the 874 female Model-versus-Control DEGs, 867 have finite fold changes in both contrasts; seven non-finite pairs were omitted from the scatter only. Opposite signs occur for 742/867 genes (85.6%), with Pearson $r = -0.596326$. The two contrasts share the Model group, so this is a descriptive pattern rather than independent evidence of molecular rescue.

Figure 6c retains the supplier hypergeometric GO/KEGG output based on the supplier default DEG list ($q \leq 0.001$ and $|\log_2$ fold-change$| \geq 1$), distinct from the $q < 0.05$ lists above. Figure 6d stars represent the supplier gene-level q values alone. The original female heatmap is retained in Fig. S18 with the original clustering. Supplementary Data 3 contains the verified source values; full supplier results are in Supplementary Data 1.

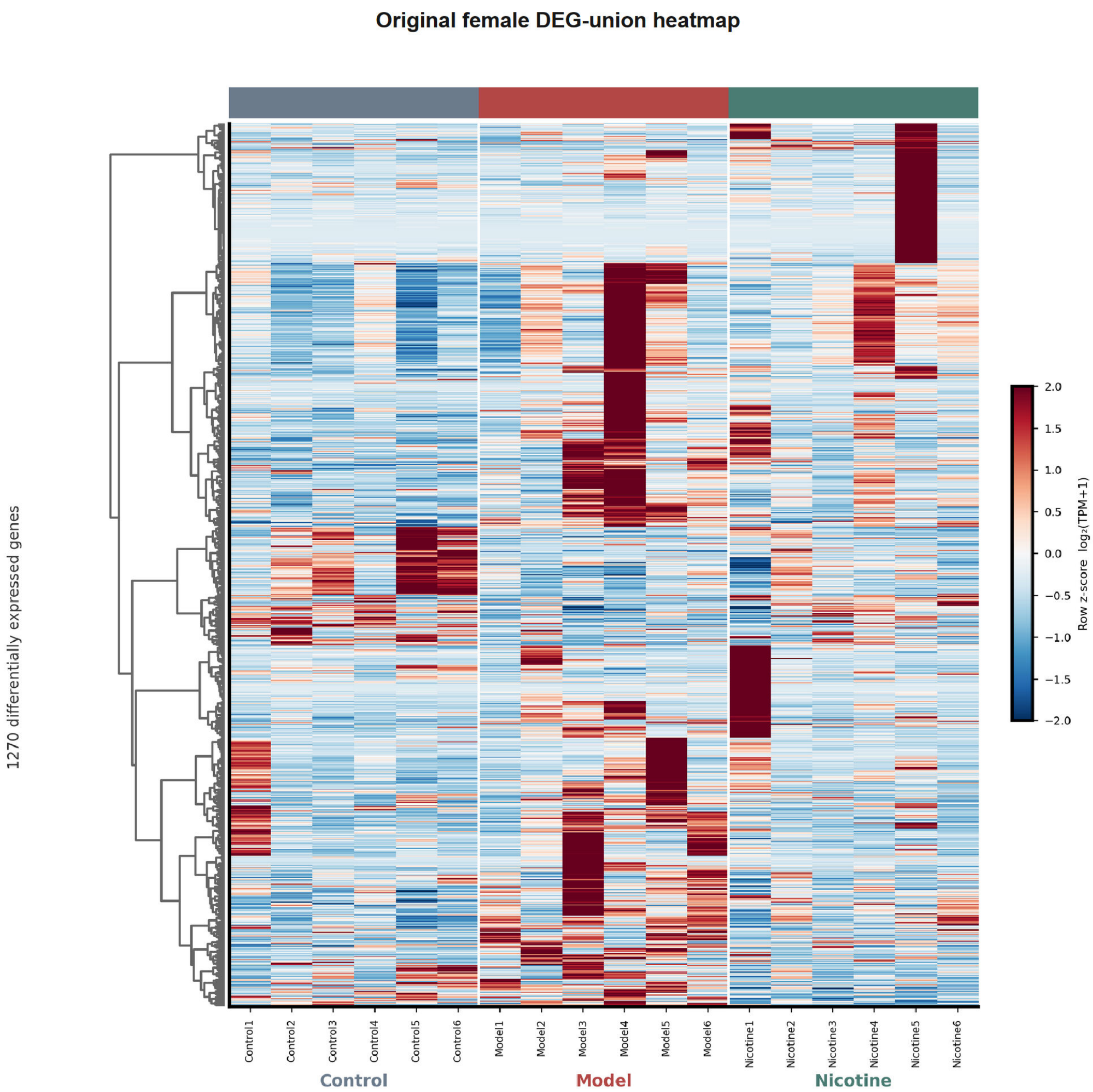

Supplementary Figure S18. Heatmap of the female hippocampal DEG union. The union comprises 1,270 genes identified in female Model-versus-Control or Nicotine-versus-Model comparisons at $q < 0.05$ and $|\log_2$ fold change$| \geq 1$. Expression is shown across 18 female WT samples, with six independent rats per treatment, as gene-wise z-scores of $\log_2(\text{TPM} + 1)$. Nicotine denotes D-galactose plus nicotine. The dendrogram indicates hierarchical clustering of the displayed genes.

Supplementary Table S15. BV2 D-galactose screening source readouts

The separate BV2 screening dataset is reported as relative assay readouts, with the mean of the zero-D-galactose column set to 1. Source column labels and all six recorded readings per level are reproduced below. Mean and SD summarize these readings descriptively; the table does not assign six independent biological replicates. This BV2 dataset is distinct from the primary donor-mouse culture experiment in Fig. 1.

Source column	Entry 1	Entry 2	Entry 3	Entry 4	Entry 5	Entry 6	Mean	SD
0	1.046192	0.985435	1.144403	0.915522	0.997087	0.911361	1.000000	0.087407
50	0.907199	0.949646	0.890553	0.916355	0.915522	0.919684	0.916493	0.019337
100	0.767374	0.762380	0.700791	0.779859	0.813150	0.755722	0.763213	0.036719
125	0.724095	0.739908	0.755722	0.771536	0.771536	0.638369	0.733528	0.050133
150	0.687474	0.732418	0.709114	0.669164	0.715772	0.719933	0.705646	0.023223
175	0.630878	0.575114	0.639201	0.464419	0.630046	0.595089	0.589125	0.065892
200	0.575114	0.547649	0.533500	0.541823	0.454432	0.560965	0.535581	0.042382
225	0.485227	0.478568	0.425302	0.386184	0.428631	0.430295	0.439034	0.037049
250	0.391178	0.356221	0.390345	0.352060	0.330420	0.329588	0.358302	0.027396
